# Optimization of an *In silico* Cardiac Cell Model for Proarrhythmia Risk Assessment

**DOI:** 10.3389/fphys.2017.00616

**Published:** 2017-08-23

**Authors:** Sara Dutta, Kelly C. Chang, Kylie A. Beattie, Jiansong Sheng, Phu N. Tran, Wendy W. Wu, Min Wu, David G. Strauss, Thomas Colatsky, Zhihua Li

**Affiliations:** ^1^Division of Applied Regulatory Science, Office of Clinical Pharmacology, Office of Translational Sciences, Center for Drug Evaluation and Research, U.S. Food and Drug Administration Silver Spring, MD, United States; ^2^Marshview Life Science Advisors Seabrook Island, SC, United States

**Keywords:** Torsade-de-Pointes (TdP), Comprehensive *in vitro* Proarrhythmia Assay (CiPA), rapid delayed rectifier potassium current (IKr), *in silico* cardiac cell model, drug block, proarrythmia risk, model optimization

## Abstract

Drug-induced Torsade-de-Pointes (TdP) has been responsible for the withdrawal of many drugs from the market and is therefore of major concern to global regulatory agencies and the pharmaceutical industry. The Comprehensive *in vitro* Proarrhythmia Assay (CiPA) was proposed to improve prediction of TdP risk, using *in silico* models and *in vitro* multi-channel pharmacology data as integral parts of this initiative. Previously, we reported that combining dynamic interactions between drugs and the rapid delayed rectifier potassium current (IKr) with multi-channel pharmacology is important for TdP risk classification, and we modified the original O'Hara Rudy ventricular cell mathematical model to include a Markov model of IKr to represent dynamic drug-IKr interactions (IKr-dynamic ORd model). We also developed a novel metric that could separate drugs with different TdP liabilities at high concentrations based on total electronic charge carried by the major inward ionic currents during the action potential. In this study, we further optimized the IKr-dynamic ORd model by refining model parameters using published human cardiomyocyte experimental data under control and drug block conditions. Using this optimized model and manual patch clamp data, we developed an updated version of the metric that quantifies the net electronic charge carried by major inward and outward ionic currents during the steady state action potential, which could classify the level of drug-induced TdP risk across a wide range of concentrations and pacing rates. We also established a framework to quantitatively evaluate a system's robustness against the induction of early afterdepolarizations (EADs), and demonstrated that the new metric is correlated with the cell's robustness to the pro-EAD perturbation of IKr conductance reduction. In summary, in this work we present an optimized model that is more consistent with experimental data, an improved metric that can classify drugs at concentrations both near and higher than clinical exposure, and a physiological framework to check the relationship between a metric and EAD. These findings provide a solid foundation for using *in silico* models for the regulatory assessment of TdP risk under the CiPA paradigm.

## Introduction

Drug-induced Torsade-de-Pointes (TdP) is a lethal arrhythmia that has caused removal of several drugs from the market (Gintant, 2008). The current cardiac safety paradigm (described by the ICH E14 and S7B guidelines) focuses on two markers to assess TdP risk: *in vitro* block of the hERG (human Ether-à-go-go-Related Gene) channel (representing the rapidly activating delayed rectifier potassium current, or IKr), and prolongation of the QTc interval in clinical studies (Sager et al., 2014). However, while eliminating the incidence of TdP in marketed drugs, this testing regime primarily aims at detecting delayed ventricular repolarization rather than the clinical end point TdP, and may be assigning proarrhythmia liability to drugs that could in fact be safe (Sager et al., 2014). Therefore, the Comprehensive *in vitro* Proarrhythmia Assay (CiPA) was proposed as a new regulatory paradigm that assesses drug TdP risk by combining measurements of drug effects on multiple cardiac ionic currents *in vitro* with *in silico* modeling of drug effects on the ventricular myocyte (Sager et al., 2014). The O'Hara Rudy cardiac cell model (ORd) (O'Hara et al., 2011) was chosen as the consensus base *in silico* model and a set of 28 drugs with known levels of clinical TdP risk (high, intermediate, low/none) were identified for the development and evaluation of the CiPA paradigm (Colatsky et al., 2016; Fermini et al., 2016). The three TdP risk categories were assigned by a Clinical Translation Working Group comprised of clinical cardiologists, safety pharmacologists, and clinical electrophysiologists according to published and publically available data and expert opinion. The 28 CiPA drugs were separated into a training set of 12 compounds to be used for calibration of the *in silico* model and the remaining 16 compounds are to be used later for validating the model. Both the training and validation compound sets comprise drugs that cover the full range of TdP risk categories and demonstrate varied electrophysiological profiles.

Previous studies have presented computational frameworks to assess TdP risk (Mirams et al., 2011; Kramer et al., 2013; Lancaster and Sobie, 2016), but their use within the CiPA framework is limited due to their differing TdP risk categories from those defined in CiPA. In addition, prior studies simulated drug effects using the half-maximal blocking concentration (IC50) for different drugs, which assumes simple pore block of the ion channels and neglects any intricacies of drug-ion channel interactions that may be important factors in predicting relative TdP risk. The importance of incorporating a kinetic representation of drug-ion channel interactions has been demonstrated in previous publications (Di Veroli et al., 2013, 2014; Li et al., 2017). In the Li et al. (2017) study we recently reported the development of a novel IKr dynamic model that can capture drug-channel dynamic interactions, and the integration of this IKr model into the ORd cardiac model with multi-channel pharmacology data. This IKr-dynamic ORd model (hereinafter referred to as the original IKr-dyn ORd model) was calibrated based on the original ORd model so that it can reproduce experimentally recorded adult human left ventricular cardiomyocyte action potential (AP) morphology and rate dependency under control (drug-free) conditions. However, this model calibration process in our previous work did not include experimental AP changes under the influence of different channel blocking drugs. This may negatively affect the model's predictive power as this model is intended for simulating drug effects under channel blocking conditions.

In this study we further optimized the original IKr-dyn ORd model by adjusting channel conductance values of major ionic currents according to human ventricular cardiomyocyte experimental data in the presence and absence of various drugs with different channel blocking activities. We show that this optimization procedure allowed the model (hereinafter referred to as optimized IKr-dyn ORd model) to quantify more accurately the impact of each individual current on the AP. We then screened a series of published and novel metrics computed by this model based on their capability of stratifying CiPA training compounds into their corresponding TdP risk categories using drug-IKr binding kinetics and multi-channel pharmacology data collected earlier through manual patch clamp systems (Li et al., 2017). The best metric identified to date is based on drug-induced changes to the net charge carried by ionic currents (qNet) during the AP, which can stratify the 12 CiPA training drugs into three TdP risk levels across various conditions. We also show that the increased predictive power of this metric is mechanistically linked to the incorporation of IKr-drug binding dynamics and the improved representation of the block effects of individual currents, two important features of the optimized IKr-dyn ORd model. Finally, we developed a framework to evaluate a cell's robustness against EAD generation, and demonstrated that the new qNet metric is correlated with the system's repolarization robustness to external pro-EAD perturbations that could reduce the membrane density of the hERG channel (IKr conductance).

## Methods

### Optimization of the IKr-dynamic ORd model

The original IKr-dyn ORd model (described in Li et al., 2017 and Expanded Methods in the Supplemental Material) was further modified (optimized IKr-dyn ORd) by scaling five ionic current conductances [IKr, the slow rectifier potassium current (IKs), inwardly rectifying potassium current (IK1), the L-type calcium current (ICaL) and the late sodium current (INaL)] so that the model provides a good fit to published APD rate dependence experimental data for control and five channel blockers (O'Hara et al., 2011). The optimization was performed using the model parameterization algorithm described in Li et al. (2014). Briefly, an initial set of scaling factors was defined within a certain range (between 0.001 and 9) and their goodness of fit was assessed using an objective cost function defined as the weighted sum of the squared errors between model simulations and experimental measurements. The set of scaling factors then underwent iterative changes (i.e., mutation and recombination) to create new generations of parameters and this process was continued until the convergence criterion was met (when the change in the minimum error of the new parameters is less than 5% over the last 30 generations). The experimental data used for fitting were taken from Figure 8 of the ORd model paper (O'Hara et al., 2011) and comprise APD rate dependence data for control and 5 drug blocking conditions: 1 μM E-4031 (70% IKr block), 1 μM HMR-1556 (90% IKs block), 1 μM nisoldipine (90% ICaL block), 100 μM BaCl2 (90% IK1 block), 10 μM mexiletine (54% INaL, 9% IKr, and 20% ICaL block). The simulated percentage of block for all drugs was kept the same as in the ORd model paper (O'Hara et al., 2011), apart from mexiletine, which used new pharmacology data from manual patch clamp systems at physiological temperatures (Crumb et al., 2016). The algorithm was run using in-house developed R scripts (R Core Team, 2014) and C programs using the Snow, Rmpi and deSolve packages (lsoda solver with a 10^−6^ relative and absolute tolerance) (Yu, 2002; Soetaert et al., 2010; Tierney et al., 2015) on the FDA High Performance Computer (HPC) with 160 cores.

### Simulation protocol for metric evaluation

All simulations were run from control steady state conditions (after 1,000 beats) at varying cycle lengths (CLs) 1,000, 2,000, and 4,000 ms and stimulus of −80 μA/μF for 0.5 ms (as in the original model). Block of ion channels at various concentrations were simulated and run for another 1,000 beats to reach a new steady state with drug. The last two beats were analyzed to check for alternans, which was observed in the presence of early afterdepolarizations (EADs), defined as having a positive derivative during the repolarization phase of the AP. The pharmacology data for the 12 CiPA training compounds (the full list and their corresponding risk categories can be found in Supplemental Table 1) were the same as in our previous report (Li et al., 2017), where drug-IKr binding kinetic parameters were estimated using an *in vitro* IKr dynamic protocol and IC50/Hill coefficients based on Crumb et al. (2016) were used for the remaining channels [the peak sodium current (INa), INaL, ICaL, IK1, IKs and transient outward potassium current (Ito); all the parameters can be found in the Supplemental Tables 2 and 3]. Simulations were run for a range of drug concentrations: from 0.5x up to 25x free maximum plasma clinical drug exposures (Cmax). Simulations were run in R and C using the deSolve package (Soetaert et al., 2010).

We assessed a range of standard metrics as also considered in Mirams et al. (2011), Lancaster and Sobie (2016): resting membrane potential (resting Vm), maximum upstroke velocity (dV/dtmax), peak membrane potential (peak Vm), APD at 50% of the amplitude (APD50), APD at 90% of the amplitude (APD90), APD triangulation (APDtri) defined as APD90-APD50, diastolic intracellular calcium concentration ([Ca^2+^]_i_) (diastolic Ca), peak [Ca^2+^]_i_ (peak Ca), calcium transient duration at 50% (CaD50) and 90% (CaD90) of the amplitude, calcium transient triangulation (Catri) defined as CaD90-CaD50, as well as the cqInward metric that quantifies the change in the amount of charge carried by INaL and ICaL, which demonstrated good separation between risk categories in our previous report (Li et al., 2017). In addition, we considered a new metric (qNet) calculated as the net charge constituting (the integral or area under the curve of) the net current (Inet) from the beginning to the end of the simulated beat (defined as Inet = ICaL + INaL + IKr + IKs + IK1 + Ito). The currents making up Inet within our study play an important role in modulating arrhythmic risk and have been chosen based on input from pharmaceutical company scientists and safety pharmacology experts as the main currents of interest within the CiPA paradigm, as outlined in Fermini et al. (2016).

To assess the robustness of a cell against EAD generation, we simulated an added perturbation by reducing the maximum conductance of IKr and reporting the minimum IKr reduction needed to trigger an EAD. Simulations were run for varying degrees of IKr conductance reduction (using a binary search algorithm) at a CL of 2,000 ms with a precision of 0.01%. For each IKr reduction tested, EADs were defined as having a positive differential (dV/dt) during the plateau phase of the AP (between APD30 and APD90) after 100 beats. The cell model was pulsed for a 100 beats before checking for EADs to allow the system to reach quasi-steady state, as in Kurata et al. (2017). The minimum IKr conductance reduction needed to trigger an EAD was named IKr reduction threshold, which reflects the system's repolarization robustness against, or distance from, EADs. To assess the relationship between the metric and the repolarization robustness, we calculated the correlation coefficients (using the pearson method) between the metric at steady state after 1,000 beats (without added IKr reduction) and the IKr reduction threshold for each drug across a series of concentrations (0.5x–25x Cmax). Situations where no IKr reduction threshold could be calculated (no EADs could be induced for the highest IKr reduction tested) or IKr reduction threshold is 0 (an EAD occurred without any added perturbation) were excluded from the correlation calculation.

### Classification methods

To assess the ability of the metrics to identify each drug's TdP risk level, we performed a proportional odds logistic regression classification and a leave-one-out validation, as in Mirams et al. (2011). If EADs were observed, the metric value at the concentration prior to EAD generation was used for the classification. We used the R lrm function from the rms package (https://CRAN.R-project.org/package=rms) and calculated the classification training error for each metric as follows: the mean (across 12 drugs) of the absolute error (difference between predicted and known risk categories), with the risk categories defined as 1 = low risk, 2 = intermediate risk and 3 = high risk. The proportional odds logistic regression model is a regression model for ordinal dependent variables, and accounts for the ordering by using cumulative probabilities defined as the odds of (Y ≤ i) = P(Y ≤ i)/(1 − P(Y ≤ i)) for each risk category i, where Y is the variable that represents a drug's risk category and P(X) is the probability of X. The function uses maximum likelihood estimates to calculate the probability of each drug being a member of each risk category, and the drug is assigned to the risk category corresponding to its highest probability. We then performed a leave-one-out validation by removing one drug from the data set and then predicting its risk category based on the classification of the remaining drugs. This was performed in turn for each drug within the data set and its leave-one-out prediction error was calculated the same way as the training error.

## Results

### Optimized IKr-dyn ORd model

**Figure 1:**
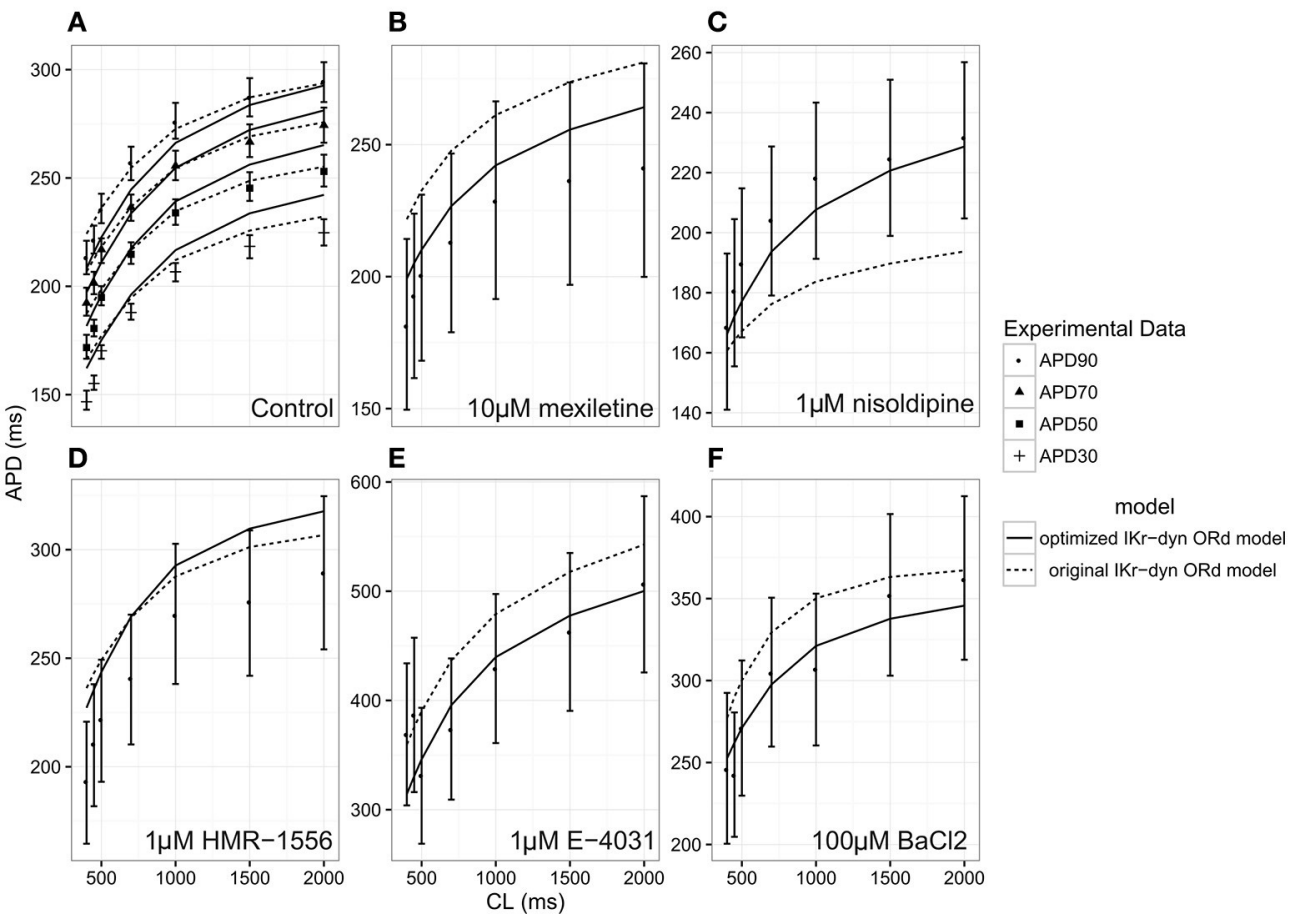
Steady state action potential duration (APD) rate dependency under the conditions of control **(A)**, 10 μM mexiletine [late sodium current INaL block **(B)**], 1 μM nisoldipine [L-type calcium current ICaL block **(C)**], 1 μM HMR-1556 [slow rectifier potassium current IKs block **(D)**], 1 μM E-4031 [rapid rectifier potassium current IKr block **(E)**] and 100 μM BACl_2_ [inwardly rectifying potassium current IK1 block **(F)**] at varying cycle lengths (CLs) for the original dynamic IKr O'Hara Rudy model (original IKr-dyn ORd; dashed lines) and the optimized dynamic IKr O'Hara Rudy model (optimized IKr-dyn ORd model; solid line). Experimental data mean (symbol) and standard deviation (error bars) are from O'Hara et al. (2011). Control **(A)** shows APD at 90% (APD90; filled circles), 70% (APD70; filled triangles), 50% (APD50; filled squares) and 30% (APD30; plus sign) repolarization. All other panels show APD90.

The optimized IKr-dyn ORd model was built by scaling the conductance of the main ion currents (IKr, IK1, IKs, INaL, ICaL) of the original IKr-dyn ORd model (presented in Li et al., 2017) to fit the APD rate dependence experimental data in control and drug block conditions from O'Hara et al. (2011). The set of scaling factors that gives the best fit for the optimized model is as follows: scaling IKr by 1.013, IKs by 1.870, IK1 by 1.698, ICaL by 1.007, and INaL by 2.661, as summarized in Table 1. A comparison of the simulation results from both the original and optimized models to the experimental data is shown in Figure 1 and the corresponding sum of squares errors (between simulation and experimental data) are shown in Table 2. Sum of squares error for the original ORd model as presented in their paper (O'Hara et al., 2011) are also shown in Table 2 for comparison purposes. We see that although for control and some current blocking conditions the original IKr-dyn ORd model has errors similar to the original ORd model, for other conditions the errors were worsened (IK1 and ICaL blocking experiments), resulting in an average error bigger than the original ORd model (72.33 vs. 57.77). However, the discrepancy between simulations and experiments was significantly reduced in the new optimized IKr-dyn ORd model.

**Table 1:** Conductance scaling factors for the original and optimized IKr-dynamic O'Hara-Rudy models (original and optimized IKr-dyn ORd): current conductances of the rapid (IKr) and slow (IKs) rectifier potassium current, inwardly rectifying potassium current (IK1), L-type calcium current (ICaL) and late sodium current (INaL) in the model are multiplied by the corresponding scaling factor.

**Scaled currents**	**Original IKr-dyn ORd model**	**Optimized IKr-dyn ORd model**
IKr	0.9	1.013
IKs	1.0	1.870
IK1	1.0	1.698
ICaL	1.0	1.007
INaL	1.0	2.661

*Note that the IKr conductance in the original IKr-dyn ORd model was scaled as described in Li et al. (2017)*.

**Table 2:** Sum of squares error (divided by 100) between experimental action potential duration (APD) rate dependence mean data (from Figure 8 in O'Hara et al., 2011) and the original O'Hara Rudy model (original ORd) (O'Hara et al., 2011), the original IKr-dyn ORd (Li et al., 2017) as well as the optimized IKr-dyn ORd under different conditions: control, mexiletine (blocks mainly INaL), HMR 1556 (blocks IKs), E-4031 (blocks IKr), BaCl2 (blocks IK1) and nisoldipine (blocks ICaL).

**Experiment**	**Sum of squares error**		
	**Original ORd**	**Original IKr-dyn ORd**	**Optimized IKr-dyn ORd**
Control	17.20	18.82	22.63
Mexiletine (INaL)	92.92^*^	91.09	18.36
HMR 1556 (IKs)	56.35	57.08	55.34
E4031 (IKr)	145.03	144.87	72.33
Bacl2 (IK1)	29.83	67.47	11.41
Nisoldipine (ICaL)	5.29	54.62	4.76
Average	57.77	72.33	30.81

* *Error was calculated using the updated mexiletine IC50 data (Crumb et al., 2016); using the block suggested in the ORd paper of 90% INaL block (O'Hara et al., 2011), the sum of squares error is of 38.48, changing the average error to 48.70*.

As can be seen in Figure 1A, under control conditions both the original IKr-dyn ORd and optimized IKr-dyn ORd models display similar behavior. Although for control data points, the optimized IKr-dyn ORd model fitting is slightly worse than the original IKr-dyn ORd model (fitting error 22.63 vs. 18.82 in Table 2), the average fit across both control and all drug block conditions is much better for the optimized IKr-dyn ORd model compared to the original IKr-dyn ORd model (fitting error 30.81 vs. 72.33). The main improvements in the quality of fit to the experimental data are observed for drug blocking conditions, especially with mexiletine (INaL blocker) and E-4031 (IKr blocker) (Figures 1B,E respectively). In the case of mexiletine, a reduction in the fitting error from 91.09 (original IKr-dyn ORd model) to 18.36 (optimized IKr-dyn ORd model) was achieved (Table 2). Figure 1B shows that, with the original IKr-dyn ORd model, the simulated APD prolongation with mexiletine is significantly longer than experimental data. A similar pattern can be seen for the IKr blocker E-4031 (Table 2 and Figure 1E). Due to the opposite roles of INaL and IKr in prolonging AP (Johannesen et al., 2016), this suggests that block of INaL is underestimated and that of IKr is overestimated in the original IKr-dyn ORd model. The optimized model corrected these inaccuracies with better fitting to the experimental data, which is important for TdP risk assessment as it is known that INaL block plays an important role in counteracting pro-arrhythmic APD prolongation of IKr block (Orth et al., 2006; Johannesen et al., 2016).

**Figure 2:**
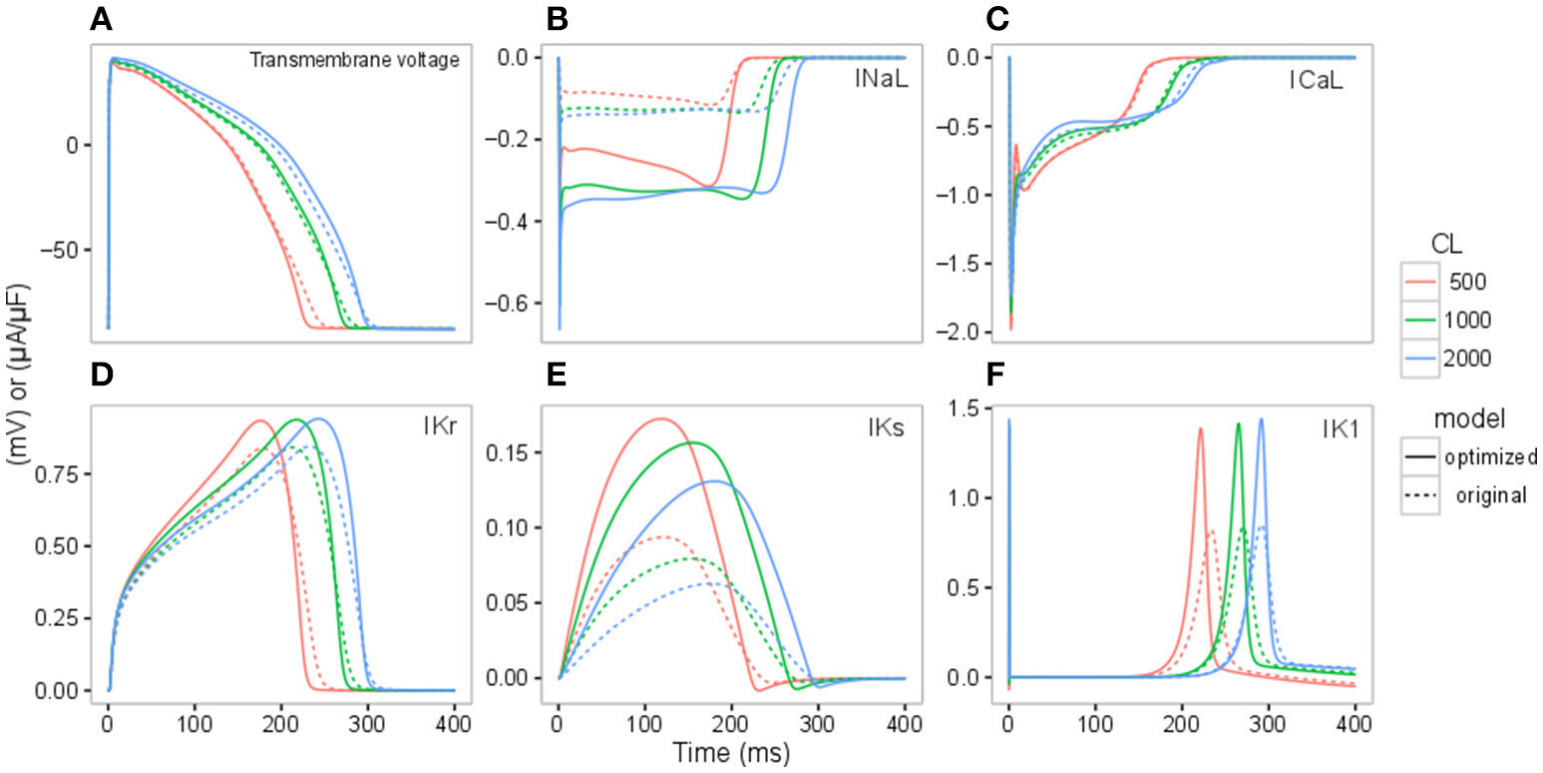
Action potential (AP) **(A)**, INaL **(B)**, ICaL **(C)**, IKr **(D)**, IKs **(E)**, IK1 **(F)** traces under control conditions for the original IKr-dyn ORd (dashed line) and the optimized IKr-dyn ORd (solid line) for CLs of 500 (red), 1,000 (green) and 2,000 (blue) ms.

To further understand the contribution of various ionic currents to AP profile after the optimization process, we compared the simulated AP traces and the ionic currents over the time course of the steady state AP between the original and our optimized IKr-dyn ORd model at different cycle lengths. As described earlier in this section all of the current conductances are increased in the optimized IKr-dyn ORd model (Table 1). However, the AP shapes from both models under control conditions are very similar, as shown in Figure 2A. This is consistent with the fact that both models fit the control AP morphology parameters (Figure 1A) reasonably well. On the other hand, while only a small change in current amplitude is observed for ICaL (Figure 2C), which only has a 0.7% change in conductance (Table 1), clear differences are observed for all other currents (IKr, INaL, IKs and IK1) with the biggest changes occurring for INaL (conductance is increased by 166.1% between the optimized and original models as shown in Table 1). This further demonstrates that INaL plays a bigger role in the optimized model than the original IKr-dyn ORd model.

### Candidate metrics

**Figure 3:**
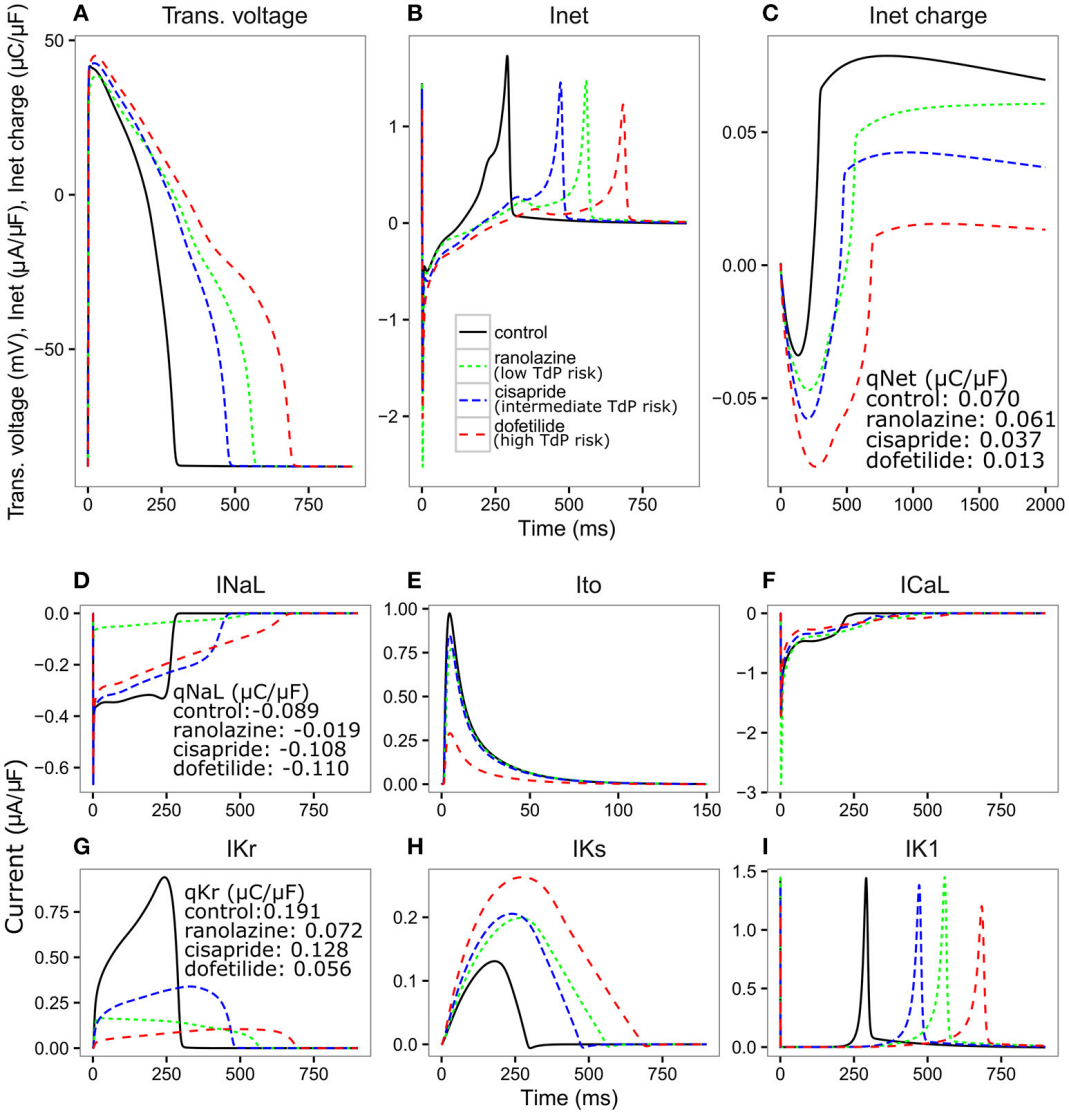
Transmembrane voltage [Trans. voltage **(A)]**, net current [Inet **(B)**], charge carried by Inet **(C)**, INaL **(D)**, Ito **(E)**, ICaL **(F)**, IKr **(G)**, IKs **(H)**, IK1 **(I)** traces for control (black solid line), ranolazine, a low TdP risk drug (green dashed line narrow spacing), cisapride, an intermediate TdP risk drug (blue dashed line normal spacing), and dofetilide, a high TdP risk drug (red dashed line wide spacing), at 25x Cmax for 2,000 ms CL using the optimized IKr-dyn ORd. Charge carried by Inet, INaL and IKr integrated from the beginning to the end of the AP beat (qX) are displayed on the graph.

We then investigated whether the optimized IKr-dyn ORd model could be used to stratify proarrhythmia risk levels. As a first step we explored the changes in AP and individual currents induced by three representative drugs (one taken from each one of the CiPA TdP risk categories), using pharmacology data as used in Li et al. (2017). Since it is known that the subtle balance between inward (such as INaL and ICaL) and outward (such as IKr, IKs, IK1, and Ito) currents underlies the generation of EADs, a mechanistic precursor to TdP (Vos et al., 1995; Volders et al., 2000; Wu et al., 2002; Weiss et al., 2010), we also examined the net current between inward and outward currents (Inet) in addition to individual currents. Figure 3 shows simulations of AP, Inet, ICaL, INaL, IKr, IKs, IK1, and Ito for ranolazine (low risk), cisapride (intermediate risk) and dofetilide (high risk), for a CL of 2,000 ms and a dose of 25x Cmax using our optimized model. A slow pacing rate (CL 2,000 ms) is used here because bradycardia is a known risk factor for TdP (Kurita et al., 1992; Kallergis et al., 2012), and a high concentration (25x Cmax) is used to highlight the potential differences between various risk levels. The amount of electronic charge carried by each current is calculated as the area under the curve (AUC) of the individual current trace and is plotted for Inet in Figure 3C.

We see in Figure 3A that all three drugs cause prolongation of APD and the low risk drug, ranolazine, shows a greater prolongation of APD compared to the intermediate risk drug, cisapride (266.78 vs. 176 ms). The performance of APD90 as a metric for all the drugs from 0.5 to 25x Cmax, can be seen in Supplemental Figure 1. In fact, verapamil and ranolazine (both low risk) display APDs greater than most intermediate risk drugs over a wide range of doses. Therefore, the amount of APD prolongation is not a good indicator of the TdP risk of a drug, demonstrating the unsuitability of APD alone as a marker for TdP risk. However, we notice that Inet (Figure 3B), calculated as the sum of the five main currents that modulate the plateau phase of the action potential (ICaL, INaL, IK1, IKr, IKs, and Ito, shown in Figures 3D–I), does correlate with the TdP risk category. As shown in Figure 3C, the order of qNet (charge carried by Inet integrated from the beginning to the end of the AP beat) is consistent with the rank order of TdP risk levels for the three drugs. At the end of the CL, ranolazine has a qNet of 0.061 μC/μF while cisapride and dofetilide have a qNet of 0.037 μC/μF and 0.013 μC/μF, respectively. A detailed examination of the individual current profiles reveals why ranolazine caused the least amount of qNet decrease. As shown in Figures 3D, G, ranolazine (green lines) caused a marked decrease of the absolute amount of charge carried by IKr (qKr decrease of 0.119 μC/μF) and INaL (qNaL decrease of 0.07 μC/μF) at the end of the AP beat compared to control (black lines). Because the outward current IKr and inward current INaL have opposite directions, ranolazine-induced reduction (in absolute values) of the two currents balanced each other and resulted in only a small change of the net charge at the end of the AP (qNet, Figure 3C). In contrast, dofetilide (Figure 3D, red lines) and cisapride (Figure 3D, blue lines) caused a significant reduction of qKr (0.135 and 0.063 μC/μF respectively) through direct channel blocking, and a slight increase of qNaL through prolonged APD. These two effects changed Inet in the same direction and worked together to decrease qNet significantly, with dofetilide causing the biggest decrease due to more significant blocking of IKr. Note that these drugs have some effects on other currents (Ito, IKs, and IK1) as well, but those changes are relatively small and will not change the rank order of qNet values significantly for the three drugs tested here. However, these other currents may become important for drugs that directly block them. For example, the effects on ICaL may be critical in determining the qNet change and risk level for a calcium blocker.

**Figure 4:**
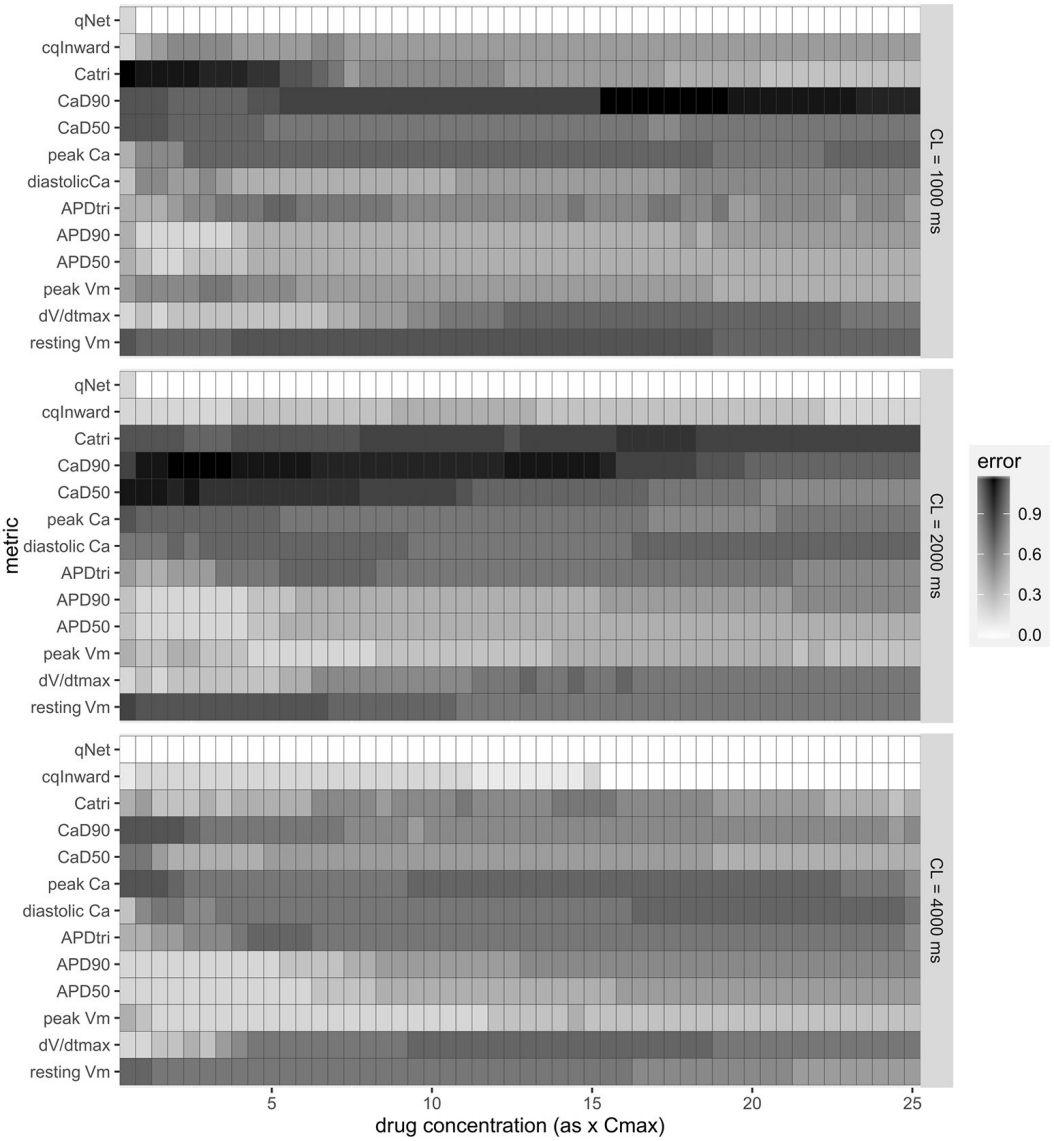
Classification training error for a range of metrics [resting membrane potential (resting Vm), maximum upstroke velocity (dV/dtmax), peak membrane potential (peak Vm), APD50, APD90, action potential (AP) triangulation (APDtri), diastolic intracellular calcium concentration ([Ca^2+^]_i_) (diastolic Ca), peak [Ca^2+^]_i_ (peak Ca), calcium transient duration at 50 and 90% of the amplitude (CaD50 and CaD90), calcium transient triangulation (Catri), change in amount of charge carried by INaL and ICaL (cqInward) and the charge carried by Inet at the end of the AP beat normalized to control (qNet)] for varying drug doses (0.5–25x Cmax) and varying CLs (1,000, 2,000, and 4,000 ms). Each box represents the mean (across 12 drugs) error (between predicted and known risk levels) for each metric at each concentration (0.5–25X Cmax). A training error of 0 represents perfect separation between the risk categories.

These initial promising results prompted us to calculate this new Inet-based metric, qNet, for all 12 CiPA training compounds and systematically compare its capability of separating the three TdP risk levels to a range of commonly tested metrics (described in the Methods section). The risk categories, IC50 and IKr dynamic parameters for each drug are listed in Supplemental Tables 1–3. Included in the comparison is also the cqInward metric, described in our previous study and defined as the normalized drug-induced change of the charge carried by the inward currents INaL and ICaL (Li et al., 2017). As shown in Figure 4, we calculated the classification training error for each metric over a range of doses (0.5–25x Cmax) and a range of CLs (1,000, 2,000, and 4,000 ms) for the 12 CiPA training compounds. This error quantifies the mean (across the 12 CiPA drugs) difference between known and predicted risk levels for each metric. We can see that across the full range of concentrations and all CLs the qNet metric shows the smallest classification training error. Notably, the qNet metric shows a classification training error of 0 for concentrations greater than or equal to 1x Cmax, meaning it consistently classifies each of the 12 CiPA training compounds into the correct TdP risk category. The cqInward metric performance is comparable to that of qNet at low pacing rates (4,000 ms) and high drug concentrations. All of the other standard metrics we considered show training errors that never come down to 0, which fluctuate across the range of doses.

The results presented in Figure 4 are consistent with the leave-one-out validation described in Table 3 performed on a subset of the doses tested (1, 10, and 20x Cmax) for a CL of 2,000 ms; the cqInward and qNet show the smallest prediction errors with values of 0.33 and 0.08 respectively at 20x Cmax. The other next best performing metrics are peak Vm with an error of 0.42 and APD50, APD90 with errors of 0.5 at Cmax 20x. Of note, at 1x Cmax, qNet and APD90 all have the same prediction error of 0.17. This is because at lower concentrations (1x Cmax) the effects of each drug are harder to differentiate due to there often being only subtle effects on the AP morphology. However, the CiPA paradigm assumes that the assessment of TdP risk may occur at any time during drug discovery and development, even prior to the time the clinical effective drug concentrations are known with any certainty. In addition, the incidence of clinical TdP is limited and not necessarily related strictly to normal (1x) clinical exposure (i.e., concomitant factors may play a role in expressing clinical TdP events). Therefore, we propose that a metric should be evaluated under multiple physiological and pharmacological conditions. The overall evidence suggest that qNet is the best among all the metrics tested, because it has a training error of 0 across a wide range concentrations (1–25x Cmax) at various pacing frequencies (2,000 and 4,000 ms), and the lowest leave-one-out error at all concentrations tested.

**Table 3:** Leave-one-out prediction error for a range of metrics at a CL of 2,000 ms and 3 doses (1, 10, and 20x Cmax): resting membrane potential (resting Vm), maximum upstroke velocity (dV/dtmax), peak membrane potential (peak Vm), APD at 50 and 90% of the amplitude (APD50 and APD90), action potential (AP) triangulation (APDtri), diastolic intracellular calcium concentration ([Ca^2+^]_i_) (diastolic Ca), peak [Ca^2+^]_i_ (peak Ca), calcium transient duration at 50 and 90% of the amplitude (CaD50 and CaD90), calcium transient triangulation (Catri), change in amount of charge carried by INaL and ICaL (cqInward) (Li et al., 2017) and charge carried by the net current (qNet).

**Metric**	**Leave-one out prediction error**		
	**1x Cmax**	**10x Cmax**	**20x Cmax**
qNet	0.17	0.08	0.08
cqInward	0.25	0.33	0.33
Catri	1.25	1.08	1.08
CaD90	1.42	1.42	0.83
CaD50	1.42	1.0	0.83
peak Ca	0.92	0.75	0.83
diastolic Ca	0.92	0.83	0.83
APDtri	0.5	0.67	0.58
APD90	0.17	0.5	0.5
APD50	0.33	0.33	0.5
peak Vm	0.42	0.42	0.42
dV/dtmax	0.42	0.83	1.17
resting Vm	1.00	0.75	0.75

### The impact of IKr-drug binding kinetics and channel conductance optimization on risk level stratification

**Figure 5:**
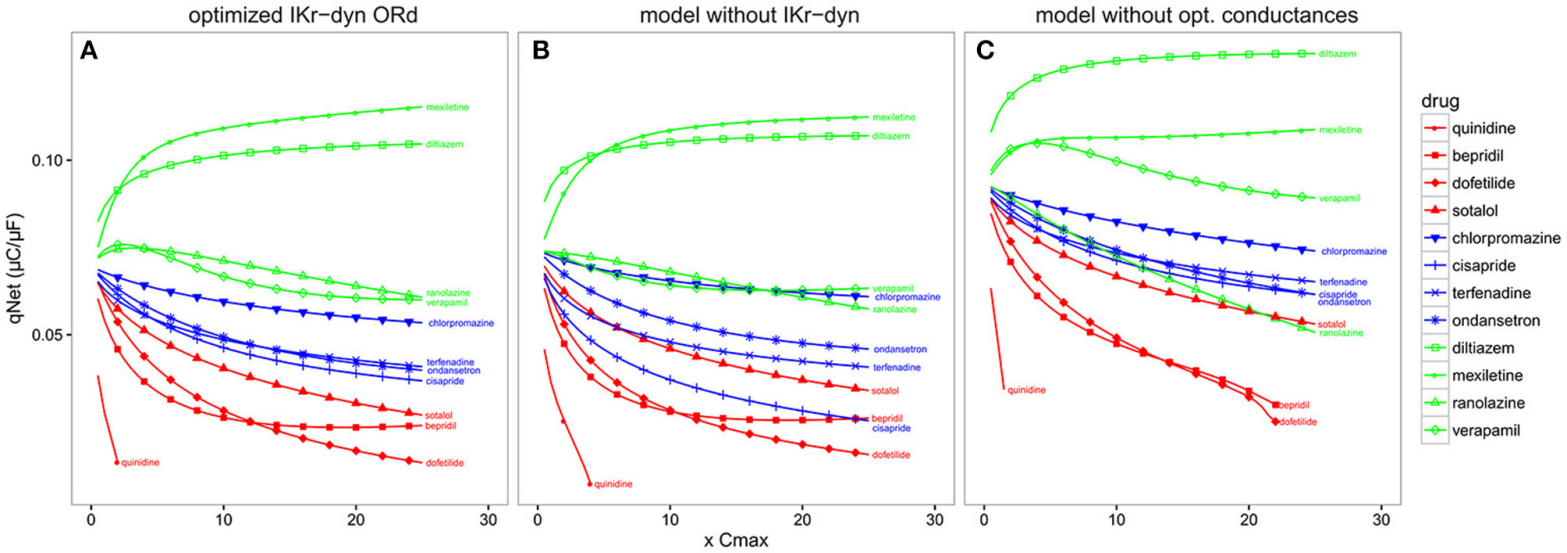
qNet for the 12 CiPA training compounds for a range of doses (0.5–25x Cmax) at a pacing rate of 2,000 ms. **(A)** Optimized IKr-dyn ORd; **(B)** A model variation without the incorporation of the IKr dynamic model (note this is the same model as in Dutta et al., 2016) and; **(C)** A model variation without the optimized channel conductances to accurately quantify block effects of individual currents (note this is the same model as in Li et al., 2017). Different TdP risk levels are color coded (high risk in red, intermediate risk in blue and low/no risk in green). Results are not shown once drug concentrations are high enough to induce early after depolarizations (EADs) (i.e., quinidine).

Compared to the original ORd (i.e., the consensus base model for CiPA), the optimized IKr-dyn ORd model presented in this work has two important changes: the incorporation of a dynamic IKr model to capture drug binding kinetics (Li et al., 2017), and an improved set of channel conductances to better represent the contribution of individual currents to AP (Figures 1, 2). In order to shed light on possible mechanistic differences among the drugs tested, we used the best candidate metric qNet as a benchmark, and compared the performance of the optimized IKr-dyn ORd model with model variations where each of the changes was removed in turn. Figure 5 shows computed qNet values for the 12 CiPA training drugs calculated over a range of drug doses from 0.5x to 25x Cmax when using the optimized IKr-dyn ORd model (Figure 5A), a model variation without incorporating the IKr dynamic model (Figure 5B) and a model variation incorporating the IKr dynamic model but without optimizing channel conductances (Figure 5C). In line with results from Figure 4 and Table 3, the metric qNet shows clear separation between the 3 TdP risk categories across the range of doses tested with the optimized IKr-dyn ORd model (Figure 5A); however, this is not the case for the other two model variations (Figures 5B,C).

The first model variation we tested does not have the IKr dynamic model incorporated but instead uses simple IC50s to represent channel block (Figure 5B). Note that this model variant has gone through a channel conductance optimization process similar to that presented in this article, as described in Dutta et al. (2016), so the difference observed between this model variant (Figure 5B) and the full optimized IKr-dyn ORd model (Figure 5A) is mainly due to the different representation of IKr block (dynamic vs. IC50s). From Figure 5B we can see that there are two intermediate risk drugs that are not correctly categorized: cisapride that is mixed with the high risk drugs, and chlorpromazine that is mixed with the low risk drugs. Cisapride is a potent and selective IKr blocker (IC50 10.1 nM and Cmax 2.6 nM see Supplementary Material), with a safety margin (IKr IC50/Cmax) of 3.8 (Redfern et al., 2003), which is close to that of the high risk drug dofetilide (IC50 4.87 nM and Cmax 2 nM, safety margin 2.4) for example. So if IC50 data are used with an assumption of simple pore drug block, cisapride is grouped with the high risk drugs. However, when we consider the IKr-drug binding dynamic data (Li et al., 2017), cisapride, but not high risk drugs like dofetilide, can rapidly dissociate from the hERG channel during diastolic intervals because it is not trapped in the closed channel. Consequently, cisapride has an actual block potency lower than high risk drugs despite similar IKr IC50/Cmax ratio, which may explain why it belongs to the intermediate rather than high risk level. On the other hand, chlorpromazine is not a potent IKr blocker (safety margin 24.4, similar to other low risk drugs) so when we look at IC50 only it is classified closer to the low risk drugs. But when IKr dynamic data are considered, chlorpromazine is highly trapped in the closed hERG channel and very slow in unbinding during diastolic intervals (Li et al., 2017). This makes it more dangerous than its IKr IC50 suggests and thus classified as an intermediate rather than low risk drug. This demonstrates that including a dynamic representation of IKr-ion channel interactions is important for categorizing TdP risk of drugs and IC50 data alone are not sufficient.

The second model variation we tested has the IKr dynamic model included, but without optimized channel conductances to reproduce AP changes under channel blocking conditions (Figure 5C). Note that this model variant is the same as the original IKr-dyn ORd model (Li et al., 2017) and, as demonstrated in Figure 1, has an inaccurate quantification of the block effects of individual currents compared to experimental data. In this scenario the low risk drug ranolazine is misclassified as a high risk compound (Figure 5C). Ranolazine is a potent IKr and INaL current blocker and these two effects can balance each other to reduce ranolazine's TdP risk (Antzelevitch et al., 2004; Johannesen et al., 2016; Saad et al., 2016). Because the INaL effect is underestimated and the IKr effect is overestimated without channel conductance optimization (Figure 1), ranolazine has a dominant IKr block effects when simulated by this model variant and thus will be mistakenly put in the high risk category (Figure 5C). Taken together, this suggests that the two added features are both important for TdP risk stratification and may mechanistically explain why a certain drug belongs to a specific TdP risk level.

### Physiological significance of qNet

In order to assess the physiological significance of the metric, we borrowed some concepts from non-linear dynamic theory, where EADs appear as membrane voltage oscillations when the equilibrium state at the plateau phase (membrane voltage between 0 and −40 mV) changes its stability via bifurcation (Qu et al., 2013; Kurata et al., 2017). The robustness of the system could be evaluated by applying a specific perturbation with a series of strengths and measuring the range of the perturbation the system can tolerate without changing stability (i.e., emergence or annihilation of oscillations) (Kurata et al., 2008). We applied this concept to our model using IKr maximum conductance reduction as a perturbation. In this case the minimum IKr reduction required to induce an EAD (IKr reduction threshold) reflects the system's robustness against, or distance from, EADs.

Therefore, for each drug over a range of concentrations from 0.5 to 25x Cmax we calculated the IKr reduction thresholds, and checked their correlation with the metrics qNet, APD90, and cqInward respectively. Detailed correlation plots for each metric can be found in the Supplemental Figures 2–4. Table 4 shows the correlation coefficients for each drug across all concentrations for IKr reduction threshold vs. qNet, APD90 and cqInward respectively. We see that qNet shows a strong correlation across all drugs (close to 1). As qNet increases the IKr reduction threshold (and the system's robustness against EAD) increases and vice versa as qNet decreases. The bigger the qNet value the safer the system is and the harder it is to induce EAD.

**Table 4:** Correlation (using pearson method) between qNet, APD90 and cqInward and IKr reduction threshold for the 11 drugs (diltiazem is not included because EADs could not be induced for the highest IKr reduction tested 99.99%) for a CL of 2,000 ms across all doses from 0.5 to 25x Cmax. Simulations where the IKr reduction threshold is 0 (EADs occur without added IKr reduction, as for quinidine ≥ 2.3x Cmax) and results where the IKr reduction threshold could not be calculated (the maximum IKr reduction tested, 99.99%, did not trigger an EAD, as for diltiazem at all Cmax, verapamil ≥ 1.7x Cmax, and mexiletine ≥ 3.8x Cmax) were excluded.

**Drug**	**Correlation with IKr reduction threshold**		
	**qNet**	**APD90**	**cqInward**
Quinidine	0.996	−0.994	−0.197
Bepridil	0.948	−0.992	0.432
Sotalol	0.979	−0.992	−0.971
Dofetilide	0.96	−0.993	−1
Cisapride	0.988	−0.996	−0.994
Ondansetron	0.997	−0.999	−0.595
Terfenadine	0.968	−0.944	0.804
Chlorpromazine	0.995	−1	0.895
Ranolazine	0.87	−0.992	0.961
Verapamil	0.977	0.998	−0.991
Mexiletine	0.974	0.989	−0.983

**Figure 6:**
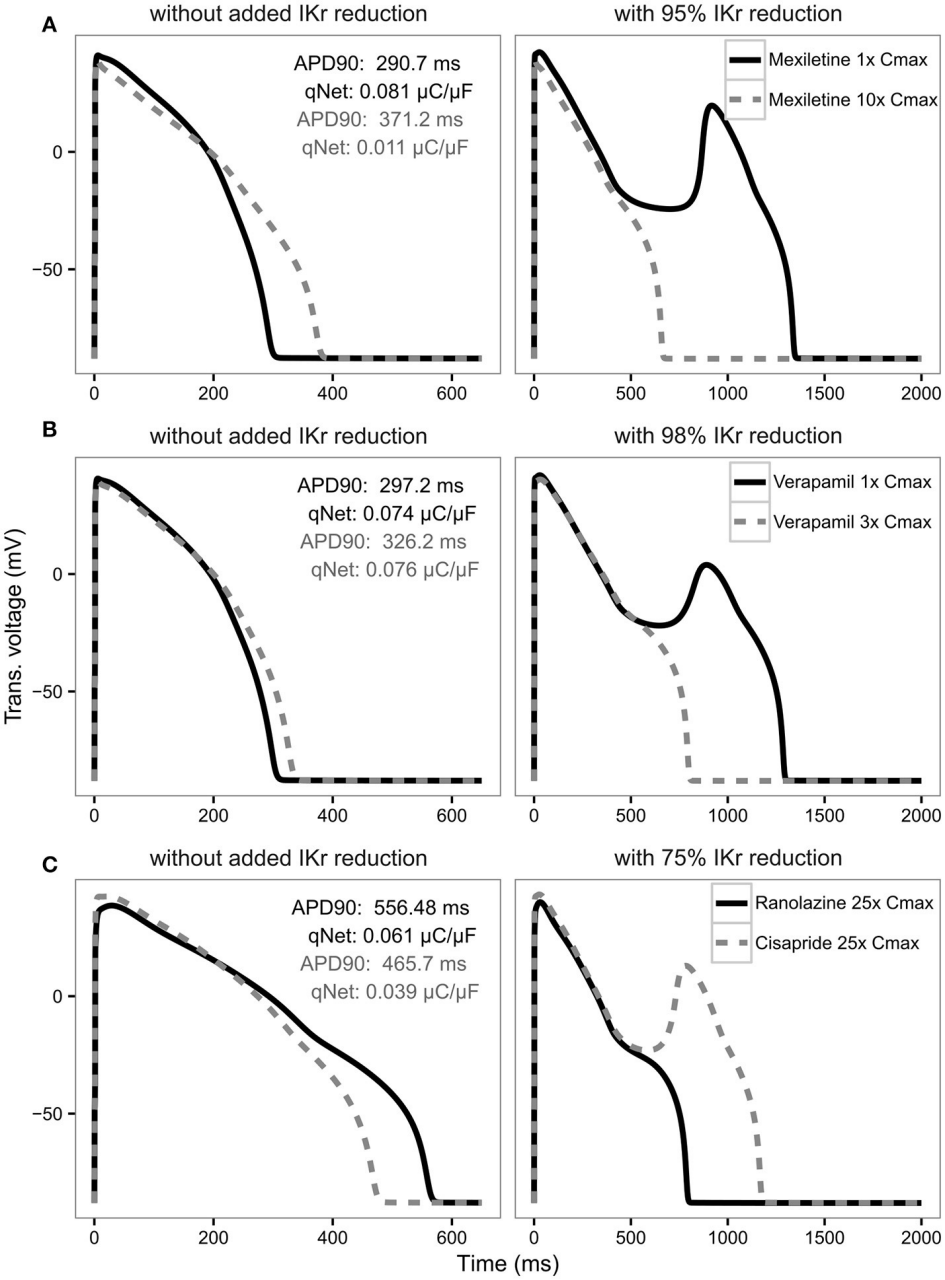
AP traces for mexiletine **(A)** at 1x Cmax (black solid line) and 10x Cmax (gray dashed line) without (left panel) and with 95% IKr reduction (right panel); verapamil **(B)** at 1x Cmax (black solid line) and 3x Cmax (gray dashed line) without (left panel) and with 98% IKr reduction (right panel); and ranolazine (black solid line) and cisapride (dashed gray line) **(C)** at 25x Cmax without (left panel) and with 75% IKr reduction (right panel) for a CL of 2,000 ms. Corresponding APD90 (ms) and qNet (μC/μF) values are reported in black for mexiletine 1x Cmax, verapamil 1x Cmax and ranolazine 25x Cmax and in gray for mexiletine 10x Cmax, verapamil 3x Cmax and cisapride 25x Cmax. Note the IKr reduction (simulated by scaling the IKr maximum conductance) is applied in addition to the drug block effect and is used to assess the system's robustness against EADs (see Results section).

For APD90, in most cases there is a strong negative correlation with IKr reduction threshold (close to −1) as expected, indicating the longer the APD the lower the repolarization robustness (i.e., the closer to EAD) and vice versa (Table 4). However, this trend reverses completely for some drugs like verapamil and mexiletine, where the correlation is positive (Table 4), suggesting the longer the APD90 the higher the repolarization robustness (the further away from EAD). This is contradictory to the general perception that longer APD90 (and QTc) signals a higher EAD/TdP liability. These unexpected relationships between APD and EAD can be seen more clearly in Figure 6, where the AP traces before and after the perturbation are shown. As can be seen from Figure 6A (left panel), using APD90 as a metric a cell under mexiletine at 1x Cmax seems safer (APD less prolonged) than at 10x Cmax, while qNet suggests otherwise (1x Cmax is more dangerous due to a smaller qNet value). When the same perturbation was applied (95% IKr reduction), the cell with 1x Cmax of mexiletine but not 10x, generated an EAD (Figure 6A right panel), indicating the cell with lower mexiletine concentration is actually closer to EAD generation, consistent with the prediction of qNet but not APD90. The same pattern can be seen in Figure 6B, where verapamil at 1x Cmax is shown to be closer to EAD than at 3x Cmax through perturbation assays (right panel), contradictory to the prediction using APD90 but not qNet (left panel). This pattern holds true when comparing ranolazine and cisapride as compared in Figure 3. As described earlier, a cell under ranolazine has a longer APD90 (indicating higher risk) and also a higher qNet value (indicating lower risk) than cisapride at 25x Cmax (Figure 6C left panel). An added perturbation of 75% IKr reduction will trigger an EAD with cisapride but not ranolazine (Figure 6C right panel), supporting the prediction of qNet but not APD90. Note that here we used 25x Cmax to match the concentrations used in Figure 3. When 1x Cmax was used, the same pattern was seen for the two drugs (see Supplemental Figure 5). This suggests under most circumstances qNet is a better metric than APD90 in marking the repolarization robustness to added perturbation of IKr reduction.

Finally, cqInward does not correlate well with robustness against EAD generation, measured as IKr reduction threshold (Table 4), despite a good performance (next to only qNet) on separating the risk categories for the training compounds (Figure 4). This suggests cqInward does not indicate the repolarization robustness to a perturbation of hERG channel density decrease. Whether cqInward is correlated with the robustness to another perturbation, or its separating power on the 12 training drugs is a non-physiological artifact, remains to be investigated. If the latter this highlights the importance to assess a metric in not only a pre-defined drug classification system, but also a physiological framework to quantitatively evaluate the correlation between the metric and EAD.

## Discussion

In this study we present an optimized version of the ORd model (O'Hara et al., 2011), which incorporates a dynamic representation of IKr to allow modeling of drug-IKr channel interactions (Li et al., 2017) as well as providing a better fit to experimental data in both control and drug blocking conditions by rescaling ionic current conductances. Most notably, INaL current is increased compared to the original model. We also demonstrate that our optimized model, used in combination with a mechanistic net charge metric (qNet), enables good separation of 12 CiPA training compounds into their respective risk categories over a range of drug concentrations and pacing rates. Furthermore, we show that this is because qNet is correlated with a system's repolarization robustness to external perturbation of hERG channel density decrease, or IKr maximum conductance reduction.

### Optimization of the O'Hara rudy model

To optimize the model we rescaled ionic current conductances in the model presented by Li et al. (2017). We demonstrate in Figure 2 that recalibration of the model conductances leads to very little changes in the AP in control conditions across a range of pacing frequencies. It did however shift the effect of some of the different currents in the cardiac cell model, most notably increasing the role of INaL in contributing to AP. A recent study also optimized the ORd model to fit various LQTS profiles (Mann et al., 2016). Mann et al. present an optimized version of the ORd model by scaling the conductances of IKs (by 5.75), IKr (by 1.00), ICaL (by 2.01), INaL (by 1.00), INaCa (by 2.95), and INaK (by 9.12). The scaling factors are different to the ones observed in our model: IKs is increased in both models although ours is increased by a smaller amount 1.87, in their model IKr is unchanged while it is slightly increased in our model, ICaL is increased significantly in their model but only very slightly in ours and INaL is unchanged in their model while it is scaled by 2.661 in our model. The differences in INaL can be explained by the differences in context of use of the model: Mann et al. investigate the effects of increased INaL (LQTS3) as opposed to drug block of INaL, as in this study. Furthermore, a key difference between our model optimization process and Mann et al. is that we used human cardiomyocyte experimental data with various channel blockers, while they used clinical LQTS data. However, one of their findings was that the ORd model over predicts the effect of IKr block (50% IKr block produced a 42% increase in APD90 as opposed to the 16.5% observed clinically), which is concurrent with our findings. An awareness of this property of the ORd model is important as the model is often considered a consensus gold standard model for simulating drug effects on cardiac cells, and properties such as the over prediction of block of IKr may lead to inaccurate predictions of drug effects on cardiac electrophysiology. Our manuscript further highlights this point and provides an alternative model with improved balance of the effect of the different ionic currents in drug block conditions.

### Performance of the qNet metric using the optimized model

Using pharmacology data for the 12 CiPA training compounds (Li et al., 2017), we assessed the suitability of a range of standard metrics based on AP morphology properties, as well as the recently published cqInward metric (Li et al., 2017) and our new qNet metric. We demonstrated that the commonly used AP-based metrics are poor indicators of TdP risk and found that our qNet metric allowed best separation of the CiPA training compounds into their risk categories. Our new metric outperformed the cqInward metric presented in Li et al. which may be a consequence of optimized channel conductances to better quantify the block effects of individual currents.

Our optimized IKr-dyn ORd model has two important features compared to the original ORd model: incorporation of modeling drug-IKr interaction kinetics based on dynamic hERG binding data (Li et al., 2017) and better characterization of individual currents' role in AP based on channel blocking data. We demonstrate the importance of simulating drug-IKr dynamics and accurate drug block conditions by providing rationale for misclassification of compounds when either one of the features were removed during TdP risk classification (Figure 5). This highlights the need for more precise model representation to simulate drug effects and stratify TdP risk levels. Additional human cardiomyocyte data may help to further refine this model.

### qNet correlates with the system's robustness against EADS

Based on ideas from non-linear dynamic theory and studies demonstrating mechanisms of EAD generation (Guo et al., 2007; Weiss et al., 2010; Xie et al., 2010; Chang et al., 2013; Kurata et al., 2017), we established a theoretical framework to quantitatively evaluate the physiological consequences of the change of the qNet (and in principle any) metric. A key concept here is the system robustness (Kurata et al., 2008), which is defined as the level of a specific perturbation the system can tolerate without a qualitative change of stability (e.g., emergence or annihilation of oscillations). We applied that concept here using IKr maximum conductance reduction as a perturbation. Note that in our model all drugs' hERG/IKr block is modeled as binding to different channel states without changing the IKr conductance. Thus the IKr conductance decrease applied here reflects extra pro-EAD perturbations independent of each drug's direct ion channel block activities, for example inter-subject variability (hERG channel density variation due to genetic background), intra-subject variability (regional difference in hERG channel density), chronic drug effects (to block hERG maturation), or drug-drug interaction. We found that qNet is correlated with the cell's repolarizing robustness to the perturbation of IKr conductance reduction. When qNet increases, the cell's IKr reduction threshold also increases, meaning the cell is moving away from EAD and needs a more severe perturbation of IKr conductance reduction to trigger an EAD. The opposite happens when qNet decreases. This positive correlation is consistent across all the compounds tested in this study. In contrast, APD90 does not show a consistent correlation with the repolarization robustness across all the drugs, suggesting for some drugs (mainly compounds with balanced inward and outward current blocking activities) APD90 may not be a good indicator of distance from EAD.

The concept of robustness to pro-EAD perturbations is highly related to that of repolarization reserve, developed by Roden (1998) to describe the redundant cellular mechanisms to effect orderly and rapid repolarization, which can be disrupted by an added stressor (perturbation), resulting in APD prolongation and/or EAD. We chose to use the term robustness instead of repolarization reserve because the latter has been widely used to describe a cell's repolarization mechanism against both delayed repolarization (APD prolongation) and voltage oscillation (EAD), which we show in Figure 6 are not necessarily correlated with each other. In contrast, robustness of a system, a concept borrowed from non-linear dynamic theory (Kurata et al., 2008), is directly related to emergence or annihilation of oscillations (EADs) in the presence of perturbations. There are different types of perturbations that could be used to test a system's robustness, for instance an applied bias current (Gray and Huelsing, 2008; Kurata et al., 2008), or an increased conductance for ICaL and/or INaL. We chose IKr conductance reduction as a perturbation because it is independent of the direct drug effects (the dynamic IKr model allows us to model IKr blockers without changing IKr conductance), and also it naturally reflects many physiological and pharmacological factors (hERG channel density variability, hERG channel trafficking block, etc.). It is possible that using different perturbations the same system can show different robustness against EADs. For example, the second best metric cqInward in terms of risk category separation does not correlate with the robustness to IKr conductance reduction, but could potentially correlate with the robustness to other perturbations. We also note that even qNet is not perfectly correlated with the robustness to IKr reduction. The correlation between qNet and IKr reduction threshold was checked only for 12 drugs at selected concentrations (0.5–25x Cmax), and it is not known if the strong correlation holds true beyond the drugs and concentrations tested. Even within the concentrations tested, some drugs (for instance ranolazine) do not have a consistent correlation across all the concentrations (Supplemental Figure 2). This suggests it may be beneficial to use the repolarization robustness (for instance IKr reduction threshold) directly as a metric so that it has clear and direct physiological meaning. However, this method is much more computationally intensive: for each drug at each concentration, the computing time for the IKr reduction threshold is more than 10 times that for qNet, as multiple levels of perturbations are needed to find the threshold. In addition, it is hard to define the metric if different perturbations to the same system lead to different thresholds (robustness). Thus, using a highly correlated surrogate metric qNet is a practical choice currently.

### Limitations and ongoing work

While the model and metric combination presented here have been able to separate all the CiPA training compounds into their respective TdP risk categories, we have yet to test this approach on the CiPA validation compounds or any compounds that were not used in the training of the model, which would provide an independent validation of this framework. A key limitation of this approach that prevents an independent validation study is that we have not provided thresholds for the qNet metric, which could be used to place an unknown compound within a specific TdP risk category. Instead we would only be able to group together compounds which would be expected to pose similar TdP risk.

As suggested in previous studies the sodium potassium pump (Lancaster and Sobie, 2016; Britton et al., 2017) and the sodium calcium exchanger (Armoundas et al., 2003; Nagy et al., 2004) play an important role in EAD generation. Simulations of hypothetical drugs by Lancaster and Sobie (2016) show that both the sodium potassium pump and sodium calcium exchanger were ranked as having the greatest influence on TdP risk, above IKs, IK1, and Ito (but excluding IKr, ICaL, and INa). Further experiments and simulations are needed to assess how CiPA drugs affect these currents and whether they should be directly taken into account in our net current calculation to improve TdP risk prediction.

Another key factor to consider is that while we have demonstrated the success of our approach using gold standard manual patch clamp data. At least in a pre-regulatory setting, the CiPA framework will likely rely on the use of high-throughput ion channel screening data acquired from different platforms routinely used within the pharmaceutical industry. We would therefore need to further refine this model to fit to high-throughput system generated data and demonstrate that the model and metric combination identified perform equally well in this case. Furthermore, dynamic modeling of other channels (such as ICaL) may be needed as the project moves forward; however, at this stage detailed kinetic drug block data for other channels is not available, nor are the protocols to extract the necessary parameters. A priority of CiPA is to keep the framework simple and constrain the cost of data generation; therefore, we use only IC50 data for other channels as, based on our current knowledge, they provide enough information to correctly separate drugs into their TdP risk categories. Additionally, calcium transient properties in the ORd model differ from other models, such as the Grandi et al. model (Grandi et al., 2010); therefore, changes to the calcium transient could improve prediction of TdP risk. In fact, Cummins et al. incorporated the Grandi et al. model [along with the ORd and the ten Tusscher et al. model (ten Tusscher and Panfilov, 2006)] in their TdP risk classification and found diastolic intracellular calcium and APD to be good markers of TdP risk (Cummins et al., 2014). However, as mentioned earlier in this study Cummins et al. define a binary TdP risk stratification that does not follow the same categorization as defined by CiPA.

A number of different avenues for further improvement of the model and TdP risk prediction approach presented here are currently being explored. We are examining the use of thresholds for TdP risk level classification, as well as incorporating both variability and uncertainty within the model predictions. In conclusion, in this manuscript we present an optimized version of the IKr-dyn ORd model presented in Li et al. (2017) that is able to accurately separate the CiPA training compounds into their respective risk categories and correlates well with the system's robustness against EADs. An independent validation of this approach is limited, but more ongoing work will see further refinement of this model and increasing its suitability to be used routinely within the CiPA paradigm.

## Author contributions

JS, PT, MW, and WW contributed to the acquisition of data and provided experimental data guidance. SD and ZL contributed to designing the work and carried out simulations. TC and DS contributed to revising the manuscript. SD, ZL, KC, and KB contributed to the analysis and interpretation of the data and writing of the manuscript.

### Conflict of interest statement

The authors declare that the research was conducted in the absence of any commercial or financial relationships that could be construed as a potential conflict of interest. The reviewer CC and handling Editor declared their shared affiliation, and the handling Editor states that the process met the standards of a fair and objective review.

## Abbreviations

CiPA: Comprehensive *in vitro* proarrhythmia assay
TdP: Torsade-de-Pointes
ORd: O'Hara Rudy dynamic cell model (O'Hara et al., 2011)
IKr-dyn ORd: ORd model with dynamic IKr
Inet: net current (sum of currents ICaL, INaL, IKr, IKs, IK1, Ito)
qNet: charge passed by Inet from the beginning to the end of the AP beat (same for qCaL and ICaL, qNaL and INaL…)
cqInward: change in charge passed by ICaL and INaL
Cmax: free maximum plasma clinical drug exposures.
