# Corrigendum: Optimization of an *In silico* Cardiac Cell Model for Proarrhythmia Risk Assessment

**DOI:** 10.3389/fphys.2017.01025

**Published:** 2017-12-06

**Authors:** Sara Dutta, Kelly C. Chang, Kylie A. Beattie, Jiansong Sheng, Phu N. Tran, Wendy W. Wu, Min Wu, David G. Strauss, Thomas Colatsky, Zhihua Li

**Affiliations:** ^1^Division of Applied Regulatory Science, Office of Clinical Pharmacology, Office of Translational Sciences, Center for Drug Evaluation and Research, U.S. Food and Drug Administration, Silver Spring, MD, United States; ^2^Marshview Life Science Advisors, Seabrook Island, SC, United States

**Keywords:** Torsade-de-Pointes (TdP), Comprehensive *in vitro* Proarrhythmia Assay (CiPA), rapid delayed rectifier potassium current (IKr), *in silico* cardiac cell model, drug block, proarrythmia risk, model optimization

In the original article, there was a mistake in Figure 6 as published. In the top left panel the qNet gray value should be 0.109 instead of 0.011. The corrected Figure 6 appears below. The authors apologize for this error and state that this does not change the scientific conclusions of the article in any way.

**Figure 6:**
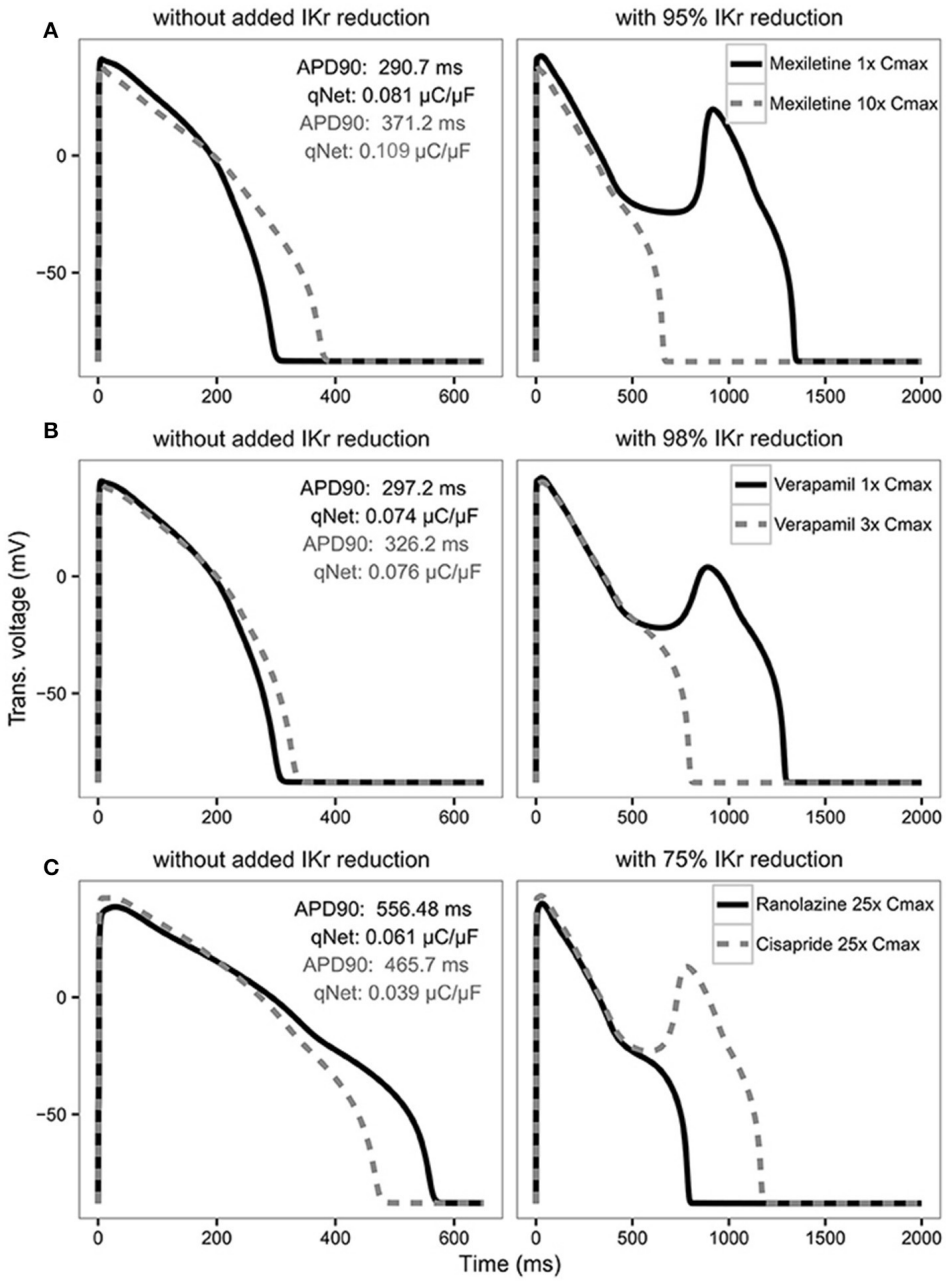
AP traces for mexiletine **(A)** at 1x Cmax (black solid line) and 10x Cmax (gray dashed line) without (left panel) and with 95% IKr reduction (right panel); verapamil **(B)** at 1x Cmax (black solid line) and 3x Cmax (gray dashed line) without (left panel) and with 98% IKr reduction (right panel); and ranolazine (black solid line) and cisapride (dashed gray line) **(C)** at 25x Cmax without (left panel) and with 75% IKr reduction (right panel) for a CL of 2,000 ms. Corresponding APD90 (ms) and qNet (μC/μF) values are reported in black for mexiletine 1x Cmax, verapamil 1x Cmax and ranolazine 25x Cmax and in gray for mexiletine 10x Cmax, verapamil 3x Cmax and cisapride 25x Cmax. Note the IKr reduction (simulated by scaling the IKr maximum conductance) is applied in addition to the drug block effect and is used to assess the system's robustness against EADs (see Results section).

## Conflict of interest statement

The authors declare that the research was conducted in the absence of any commercial or financial relationships that could be construed as a potential conflict of interest.

