# Supplementary material for: Optimization of an In silico Cardiac Cell Model for Proarrhythmia Risk Assessment
Source: Front Physiol. 2017 Aug 23;8:616. doi: 10.3389/fphys.2017.00616 (PMC5572155; doi:10.3389/fphys.2017.00616)
Supplement: Supplementary file 1 [file Presentation1.pdf]

Supplemental material

Expanded Methods: Detailed description of the dynamic IKr model (Li et al., 2017)

The dynamic IKr model within the optimized IKr-dyn ORd model was the same as described in our previous paper and the details can be found there (Li et al. 2017). Briefly, it contains a physiological component representing channel gating and a pharmacodynamic component representing drug binding. The physiological component is based on our recently published temperature-dependent hERG model (Li et al., 2016) and further optimized to recapitulate native IKr behavior. It has two closed states (C), two inactivated-closed states (IC), one open state (O), and one inactivated-open state (IO). The pharmacodynamic component has three drug-bound states: open-bound (O*), closed-bound (C*) and open-inactivated-bound (IO*). Ku is the unbinding reaction (O*→O) rate while the binding reaction rates (O→O* and IO→IO*) are equal to Ku*Emax(D), where Emax is a sigmoid model describing the concentration-response of each drug and D is the drug concentration in nM. The Emax model is defined as $(\mathrm{Kmax} \cdot \mathrm{D}^{\mathrm{n}})/(\mathrm{D}^{\mathrm{n}} + \mathrm{EC50}^{\mathrm{n}})$, where Kmax is the maximum drug effect at saturating concentrations, D is the concentration/dose of the drug, n is the Hill coefficient, and EC50 is the concentration when half maximum effect is achieved. The "trapping rate" (Kt) of channel closing with drug bound (IO*→C* and O*→C*) was manually fixed at $3.5 \cdot 10^{-5}\ \mathrm{ms}^{-1}$. The rate of channel opening when drug is bound (C*→O* and C*→IO*) is equal to $\mathrm{Kt} \cdot \mathrm{X(V)}$, where X is the steady-state gate activation function for IKr in ORd model (O'Hara et al., 2011) and V is the transmembrane potential in millivolts (mV). X(V) is defined as $1/\left(1 + \exp(-(\mathrm{V} - \mathrm{Vhalf\text{-}trap})/6.789)\right)$, where Vhalf-trap is the membrane voltage at which half of drug-bound channels are open. The IO* to IO transition rate is determined by microscopic reversibility. Some important considerations while developing this model are listed below.

1. IC50 vs. EC50

A Hill equation is often used to describe the concentration-dependent steady state fraction of block and the IC50 is the drug concentration where the steady-state inhibition reaches 50% of maximally possible block level. On the other hand, in the field of mechanism-based pharmacodynamics modeling a Hill equation-like Emax format is often used to describe concentration-dependent reaction rate (Felmlee et al., 2012) where EC50 is the drug concentration when the reaction rate reaches 50% of the maximum speed. We applied the Emax format to concentration-dependent binding rates governing the transitions between open (O) and inactivated open (IO) to open bound (O*) and inactivated open bound (IO*) (O→O* and IO→IO*). Therefore, EC50 and IC50 describe different concepts despite similar names and similar format of equations (Hill-like equations).

2. Unusually high EC50 values

When the EC50 is small (close to concentrations tested experimentally), the Emax equation used is the bona fide Emax model (i.e. the relationship between drug concentration and reaction rates O→O* or IO→IO* is sigmoidal with a saturating effect). When the EC50 is much larger than the highest concentration tested then the Emax model becomes a liner model because $\mathrm{Kmax}/(\mathrm{D}^{\mathrm{n}} + \mathrm{EC50}^{\mathrm{n}}) \approx \mathrm{Kmax}/\mathrm{EC50}^{\mathrm{n}}$. In this case the ratio $\mathrm{Kmax}/\mathrm{EC50}^{\mathrm{n}}$ describes the linear relationship between drug concentration and reaction rates for O→O* or IO→IO*. Under this "linear approximation" circumstances the two parameters Kmax and $\mathrm{EC50}^{\mathrm{n}}$ are unidentifiable. However, it's their ratio that really matters, and the ratio is actually identifiable via a global identifiability test (Table S2 in (Li et al., 2017)): consistent ratio values were obtained from repeated fitting runs with different starting points (random seeds).

3. The choice of Emax model

Currently, the intended purpose of this model within a regulatory pipeline (Comprehensive in vitro Proarrhythmia Assay), constrains all new compounds to be evaluated/simulated by the same model. Therefore, the drug binding component of this model needs to be flexible enough to represent different mode of binding, for example linear vs Emax form of binding rate. Because most current drug-hERG dynamic models assumes only linear relationship between the drug concentration and binding rate, we first tried a linear form of binding rate where the reaction rates O→O* or IO→IO* are of the form $\mathrm{Kf} \cdot \mathrm{D}$ (Kf is diffusion rate while D is drug concentration). We found that a much worse fit was achieved for some compounds when assuming the linear drug block model (data not shown). We think it is partly because some drugs (like cisapride) have a saturating effect in its concentration-binding rate relationship, and only a model like Emax could capture that. Therefore, the current Emax formula was chosen to accommodate both a bona fide Emax model (with suturing effect) and (through linear approximation) a linear model (no saturating effect).

4. The choice of Kt value

When we developed the model a key "reality check" is to reproduce different drugs' ability to cause reverse use dependency (RUD) at APD prolongation (Fig 5 of our previous paper (Li et al., 2017)). We found that when allowing the Kt parameter to vary (through fitting) the RUD pattern is incorrect: dofetilide may have no RUD while verapamil may have it, which is contradictory to many published data. The value Kt was therefore fixed and was selected to best replicate these reported RUD patterns.

5. Number of bound states

An important consideration for CiPA is ease of implementation; therefore, we decided to find the simplest drug binding model to explain the data. The simplest drug binding format in the literature, which is widely used, is assuming two drug-bound states: open-bound and inactivated-

bound, corresponding to O* and IO*. However, it didn't work well, probably because it didn't capture the "trapping" phenomenon (Li et al., 2017). After that we found the current drug bound model (three drug-bound states O*, IO*, and C*) was the simplest addition to the base IKr model which was able to provide the best fit to the fractional block data. While we could have explored the use of a more complex model including additional closed and inactivated bound states this would have increased the complexity of the model structure so we selected the simpler drug bound component with just one open, inactivated and closed state.

Supplemental Tables

The following Supplemental Tables are a reproduction of Tables 1, 2 and 3 from (Li et al., 2017). They are included here to give more context about the current work.

Supplemental Table 1. The 12 CiPA training compounds.

Drug	indication	CiPA TdP Category
quinidine	antiarrhythmic	High
bepridil	angina	High
dofetilide	antiarrhythmic	High
sotalol	antiarrhythmic	High
chlorpromazine	antipsychotic	Intermediate
cisapride	gastrokinetic	Intermediate
terfenadine	antihistamine	Intermediate
ondansetron	antimimetic	Intermediate
diltiazem	hypertension/angina	Low/No Risk
mexiletine	antiarrhythmic	Low/No Risk
ranolazine	angina	Low/No Risk
verapamil	hypertension/angina	Low/No Risk

Supplemental Table 2. IKr-dynamic binding parameters for all CiPA training compounds and the therapeutic free plasma concentrations (Free Cmax) used in the simulations. For most drugs, the estimated $EC50^n$ >> concentrations tested, and the Emax model used to describe dose-response relationship essentially becomes a linear model (see (Li et al., 2017)). For other drugs like cisapride and diltiazem, the estimated $EC50^n$s are not significantly higher than the tested concentrations ($EC50^n$ < 10-fold of estimated IC50), suggesting there is indeed a sigmoidal dose-response relationship. The free Cmax values are either directly taken from the referenced paper, or in the case of ondansetron, calculated using reported total Cmax and protein binding data from FDA drug labels.

drug	Kmax	Ku (ms $^{-1}$)	EC50^{n} (nM)	n	Vhalf-trap (mV)	Free Cmax (nM)
quinidine	5770	0.01	1.00E+06	0.8311	-64.87	3237(Redfern et al., 2003)
bepridil	37350000	0.0001765	1.00E+09	0.9365	-54.93	33(Redfern et al., 2003)
dofetilide	1.00E+08	1.79E-05	548300000	0.9999	-1.147	2(Redfern et al., 2003)
sotalol	2403	0.01985	9619000	0.7516	-55	14690(Kramer et al., 2013)
chlorpromazine	206000	0.03866	56770000	0.8871	-14.57	38(Redfern et al., 2003)
cisapride	9.997	0.0004161	42.06	0.9728	-199.5	2.6(Crumb et al., 2016)
terfenadine	9884	8.18E-05	41380	0.65	-77.49	4(Redfern et al., 2003)
ondansetron	33540	0.02325	9950000	0.8874	-82.11	139
diltiazem	251	0.2816	1.00E+06	0.9485	-90.89	122(Redfern et al., 2003)
mexiletine	9.996	0.09967	2308000	1.304	-86.26	4129(Redfern et al., 2003)
ranolazine	55.84	0.01929	147200	0.95	-94.87	1948.2(Crumb et al., 2016)
verapamil	46460	0.0007927	9184000	1.043	-100	81(Redfern et al., 2003)

Supplemental Table 3. IC50s used in AP simulation.

IC50s and Hill coefficients (h's) for IKr are estimated using Milnes protocol data in this study (Milnes et al., 2010; Li et al., 2017). Other channels' IC50 and h values are calculated using the blocking data from Crumb et al. (Crumb et al., 2016). Zeros in the table indicate no detectable blocking for that channel. Note that many drugs' IC50s are very high because only a narrow concentration range around Cmax was tested in Crumb study (Crumb et al., 2016). In those cases, the estimated IC50s may not be accurate in predicting block at high concentrations. Therefore, all simulations in this study were limited to ≤25x Cmax.

drug		hERG	INaL	ICaL	INa	Ito	IK1	IKs
quinidine	IC50 (nM)	992	9417	51592.3	12329	3487.4	39589919	4898.9
quinidine	h	0.8	1.3	0.6	1.5	1.3	0.4	1.4
bepridil	IC50 (nM)	50	1813.9	2808.1	2929.3	8594	0	28628.3
bepridil	h	0.9	1.4	0.6	1.2	3.5	0	0.7
dofetilide	IC50 (nM)	4.9	753160.4	260.3	380.5	18.8	394.3	0
dofetilide	h	0.9	0.3	1.2	0.9	0.8	0.8	0
sotalol	IC50 (nM)	110600	0	7061527	1.14E+9	43143455	3050260	4221856
sotalol	h	0.8	0	0.9	0.5	0.7	1.2	1.2
chlorpromazine	IC50 (nM)	929.2	4559.6	8191.9	4535.6	17616711	9269.9	0
chlorpromazine	h	0.8	0.9	0.8	2	0.4	0.7	0
cisapride	IC50 (nM)	10.1	0	9258076	0	219112.4	29498	81192862
cisapride	h	0.7	0	0.4	0	0.2	0.5	0.3
terfenadine	IC50 (nM)	23	20056	700.4	4803.2	239960.8	0	399754
terfenadine	h	0.6	0.6	0.7	1	0.3	0	0.5
ondansetron	IC50 (nM)	1320	19180.8	22551.4	57666.4	1023378	0	569807
ondansetron	h	0.9	1	0.8	1	1	0	0.7
diltiazem	IC50 (nM)	13150	21868.5	112.1	110859	2.82E+09	0	0
diltiazem	h	0.9	0.7	0.7	0.7	0.2	0	0
mexiletine	IC50 (nM)	28880	8956.8	38243.6	0	0	0	0
mexiletine	h	0.9	1.4	1	0	0	0	0
ranolazine	IC50 (nM)	8270	7884.5	0	68774	0	0	36155020
ranolazine	h	0.9	0.9	0	1.4	0	0	0.5
verapamil	IC50 (nM)	288	7028	201.8	0	13429.2	3.49E+8	0
verapamil	h	1	1	1.1	0	0.8	0.3	0

Supplemental Figures

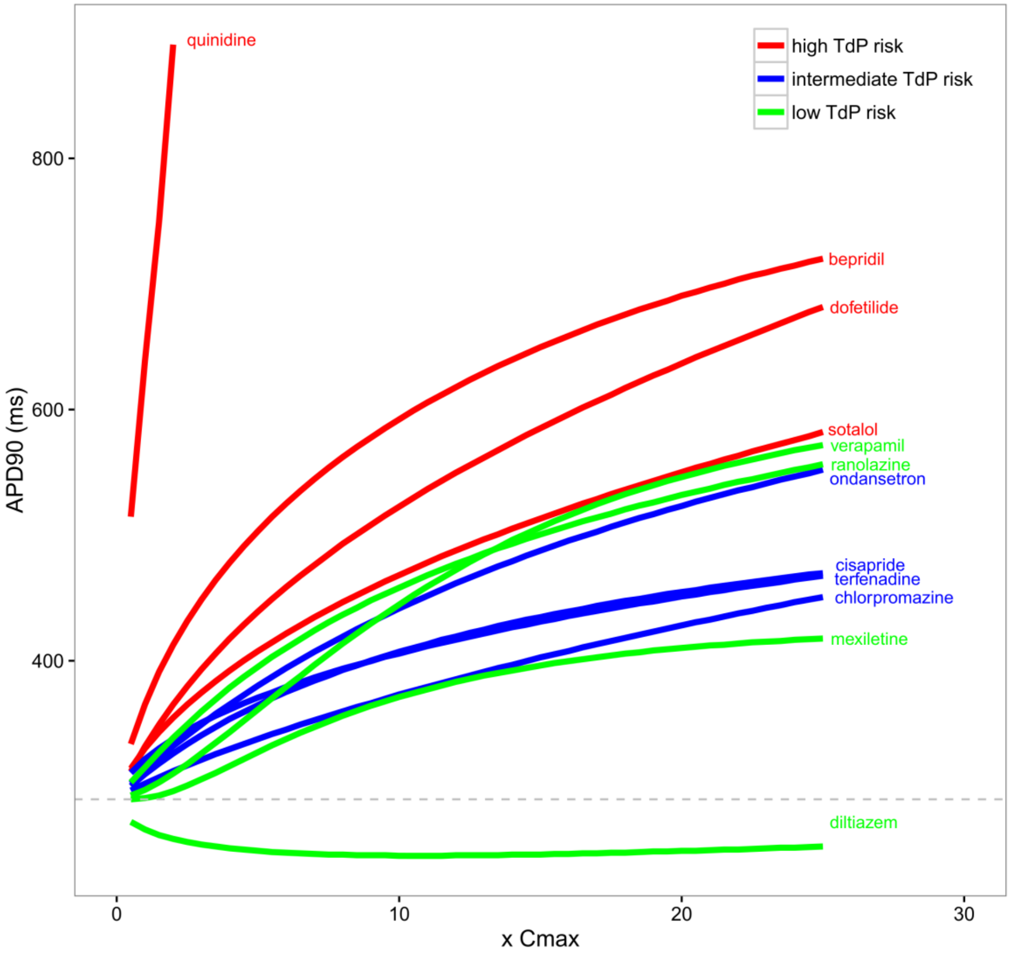

Supplemental Figure 1: Action potential duration at 90% repolarization (APD90) for 0.5x to 25x Cmax. Different TdP risk levels are color coded (high risk in red, intermediate risk in blue and low/no risk in green). Results are not shown once drug concentrations are high enough to induce EADs (i.e. quinidine). Gray dashed line represents APD90 without drug.

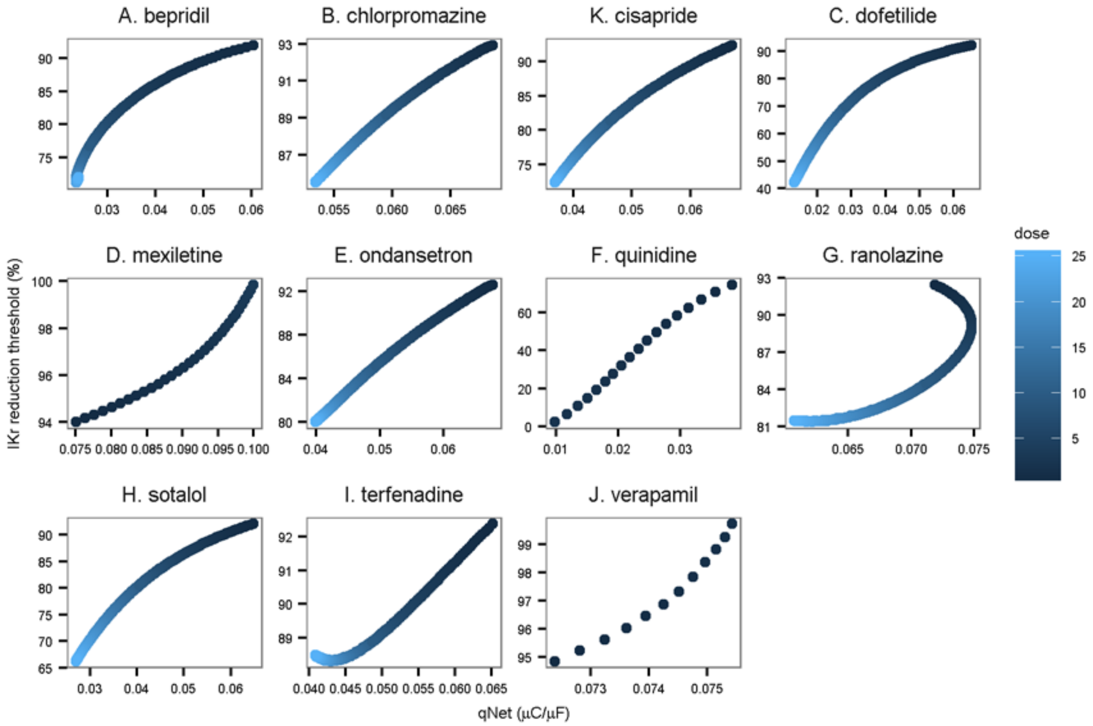

Supplemental Figure 2: Relationship between the IKr reduction threshold and the charge passed by the net current (qNet) for 0.5x (dark blue) to 25x (light blue) Cmax for each drug. Results where EADs occur without added IKr reduction (quinidine ≥ 2.3x Cmax) and results where the maximum IKr reduction did not trigger an EAD (diltiazem all Cmax; verapamil ≥ 1.7x Cmax and mexiletine ≥ 3.8x Cmax) were excluded. See Table 4 (main text) for correlation coefficients.

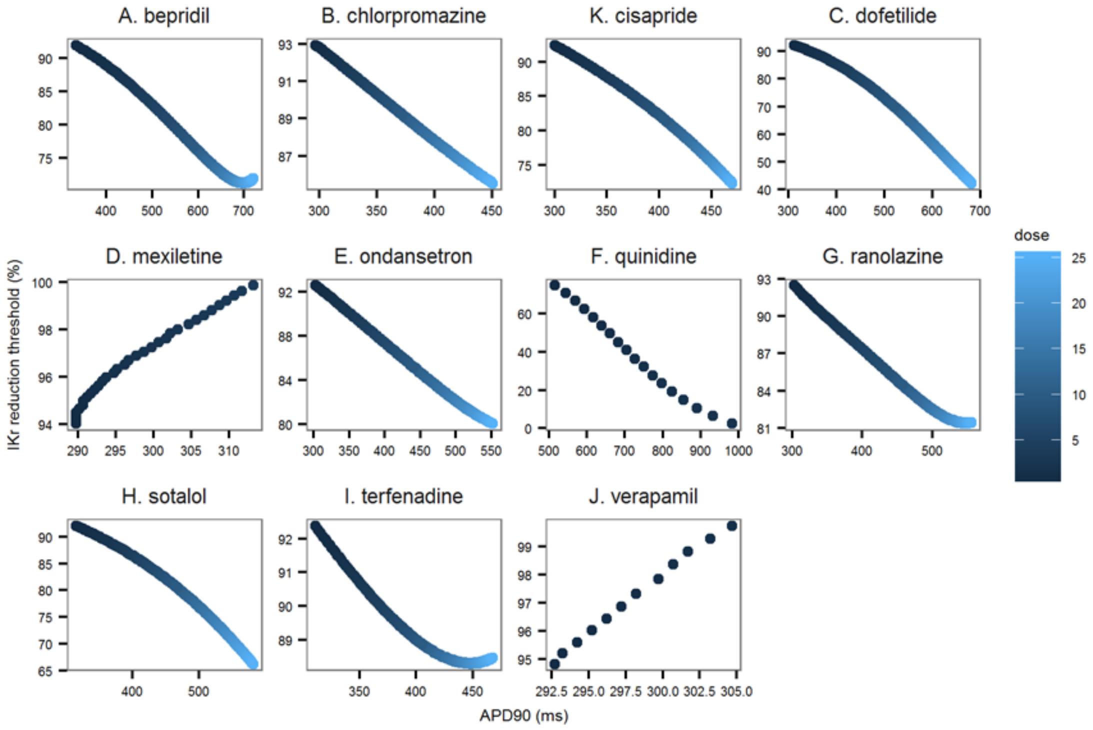

Supplemental Figure 3: Relationship between the IKr reduction threshold and APD90 for 0.5x (dark blue) to 25x (light blue) Cmax for each drug. Results where EADs occur without added IKr reduction (quinidine ≥ 2.3x Cmax) and results where the maximum IKr reduction did not trigger an EAD (diltiazem all Cmax; verapamil ≥ 1.7x Cmax; mexiletine ≥ 3.8x Cmax) were excluded. See Table 4 (main text) for correlation coefficients (Table 4 shows that mexiletine and verapamil have positive correlation coefficients as opposed to negative correlation coefficients for all other drugs).

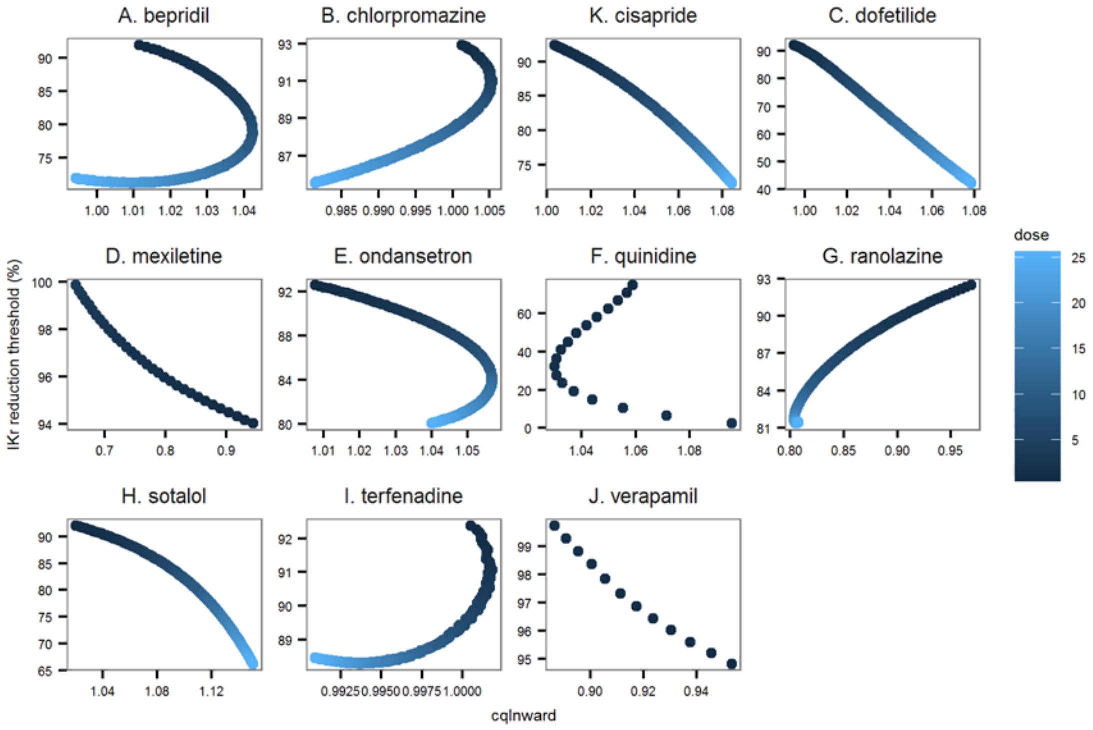

Supplemental Figure 4: Relationship between the IKr reduction threshold and the normalized charge passed by INaL and ICaL (cqInward (Li et al., 2017)) for 0.5x (dark blue) to 25x (light blue) Cmax for each drug. Results where EADs occur without added IKr reduction (quinidine ≥ 2.3x Cmax) and results where the maximum IKr reduction did not trigger an EAD (diltiazem all Cmax; verapamil ≥ 1.7x Cmax; mexiletine ≥ 3.8x Cmax) were excluded. See Table 4 (main text) for correlation coefficients.

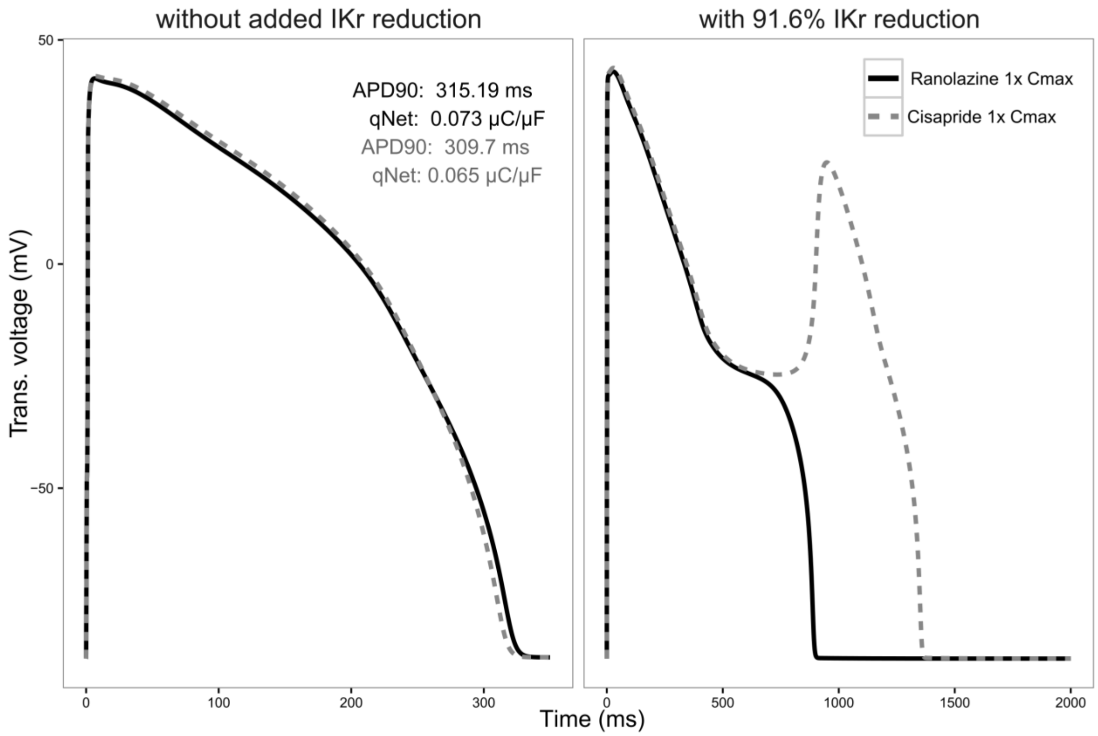

Supplemental Figure 5: Transmembrane voltage (Trans. voltage) through time for ranolazine (black solid line) and cisapride (dashed gray line) at 1x Cmax without (left panel) and with 91.6% IKr reduction (right panel) for a CL of 2000 ms. Corresponding APD90 (ms) and qNet (μC/μF) are reported in black for ranolazine and in gray for cisapride. Note the IKr reduction (simulated by scaling the IKr maximum conductance) is applied in addition to the drug block effect and is used to assess the system's robustness against EADs (see Results section).

References

Crumb, W.J., Jr., Vicente, J., Johannesen, L., and Strauss, D.G. (2016). An evaluation of 30 clinical drugs against the comprehensive in vitro proarrhythmia assay (CiPA) proposed ion channel panel. *J Pharmacol Toxicol Methods*. doi: 10.1016/j.vascn.2016.03.009.

Felmlee, M.A., Morris, M.E., and Mager, D.E. (2012). Mechanism-based pharmacodynamic modeling. *Methods Mol Biol* 929**,** 583-600. doi: 10.1007/978-1-62703-050-2_21.

Kramer, J., Obejero-Paz, C., Myatt, G., Kuryshev, Y., Bruening-Wright, A., Verducci, J., et al. (2013). MICE Models: Superior to the HERG Model in Predicting Torsade de Pointes. *Scientific Reports* 3. doi: doi: 10.1038/srep02100.

Li, Z., Dutta, S., Sheng, J., Tran, P., Wu, W., and Colatsky, T. (2016). A temperature-dependent in silico model of the human ether-à-go-go-related (hERG) gene channel. *Journal of Pharmacological and Toxicological Methods*. doi: doi: 10.1016/j.vascn.2016.05.005.

Li, Z., Dutta, S., Sheng, J., Tran, P.N., Wu, W., Chang, K., et al. (2017). Improving the In Silico Assessment of Proarrhythmia Risk by Combining hERG (Human Ether-à-go-go-Related Gene) Channel–Drug Binding Kinetics and Multichannel Pharmacology. *Circulation: Arrhythmia and Electrophysiology* 10(2)**,** e004628.

Milnes, J., Witchel, H., Leaney, J., Leishman, D., and Hancox, J. (2010). Investigating dynamic protocol-dependence of hERG potassium channel inhibition at 37°C: Cisapride versus dofetilide. *Journal of Pharmacological and Toxicological Methods* 61(2)**,** 178-191. doi: doi: 10.1016/j.vascn.2010.02.007.

O'Hara, T., Virág, L., Varró, A., and Rudy, Y. (2011). Simulation of the undiseased human cardiac ventricular action potential: model formulation and experimental validation. *PLoS computational biology* 7(5)**,** e1002061. doi: doi: 10.1371/journal.pcbi.1002061.

Redfern, W.S., Carlsson, L., Davis, A.S., Lynch, W.G., MacKenzie, I., Palethorpe, S., et al. (2003). Relationships between preclinical cardiac electrophysiology, clinical QT interval prolongation and torsade de pointes for a broad range of drugs: evidence for a provisional safety margin in drug development. *Cardiovascular Research* 58(1)**,** 32-45. doi: S0008636302008465.
